# Cardiovascular outcomes of SGLT2 inhibitors versus GLP-1 receptor agonists in patients with type 2 diabetes and a history of myocardial infarction: a systematic review and network meta-analysis of randomized controlled trials

**DOI:** 10.3389/fendo.2026.1902802

**Published:** 2026-07-29

**Authors:** Yanxia Ren, Hui Yang, Shuang Zhang, Tianxiao Hu, Qingying Tan, Jing Wang, Yao Xu

**Affiliations:** 1 Department of Endocrinology and Metabolism of the 903rd Hospital of the Joint Logistic Support Force, Hangzhou, China; 2 Cardiovascular Department 2 of the First Affiliated Hospital of Shihezi University, Xinjiang, China; 3 Department of Burn Plastic Surgery of the 903rd Hospital of the Joint Logistic Support Force, Hangzhou, China

**Keywords:** cardiovascular outcomes, GLP-1 receptor agonists, heart failure, major adverse cardiovascular events, meta-analysis, myocardial infarction, SGLT2 inhibitors, type 2 diabetes

## Abstract

**Background:**

Patients with type 2 diabetes (T2DM) and a history of myocardial infarction (MI) face a high risk of recurrent cardiovascular events. Sodium-glucose cotransporter-2 inhibitors (SGLT2i) and glucagon-like peptide-1 receptor agonists (GLP-1 RAs) reduce cardiovascular risk, but no head-to-head trials exist. We performed a systematic review and network meta-analysis (NMA) to indirectly compare their cardiovascular efficacy in this high-risk population.

**Methods:**

Randomized controlled trials (RCTs) published up to February 1, 2026, were systematically searched and evaluated. The primary outcome was major adverse cardiovascular events (MACE); secondary outcomes included hospitalization for heart failure (HFH), MI, and cardiovascular death (CV death). A frequentist NMA was used as the primary comparative analysis, complemented by Bucher interaction tests as sensitivity analyses. Evidence certainty was assessed with GRADE.

**Results:**

Eleven RCTs were included; after exclusion of overlapping populations, the final analyses comprised 7 studies for MACE (2 SGLT2i, 5 GLP-1 RA), 6 for HFH (2 SGLT2i, 4 GLP-1 RA), 4 for MI (1 SGLT2i, 3 GLP-1 RA), and 6 for CV death (2 SGLT2i, 4 GLP-1 RA). Both drug classes significantly reduced MACE compared to placebo (SGLT2i HR 0.87, 95% CI 0.77–0.97; GLP-1 RA HR 0.83, 95% CI 0.77–0.89). The NMA indirect comparison for MACE yielded an HR of 1.05 (95% CI 0.92–1.20, p = 0.44), indicating no statistically significant difference between classes. For HFH, SGLT2i exhibited a numerically greater reduction (indirect HR 0.83, 95% CI 0.67–1.03, p = 0.09) that did not reach conventional significance. MI risk was similarly reduced by both classes (indirect HR 1.05, 95% CI 0.83–1.33, p = 0.68). For CV death, the indirect comparison showed no statistically significant difference between classes (HR 0.94, 95% CI 0.76–1.16, p = 0.57), but this estimate is based on limited SGLT2i data (only two trials). The GRADE certainty was low for CV death, primarily due to indirectness (predominantly remote MI populations) and imprecision (wide confidence intervals crossing the line of no effect).

**Conclusions:**

In patients with T2DM and a history of MI, indirect evidence suggests that both SGLT2i and GLP-1 RAs reduce MACE, HFH, and recurrent MI, with no statistically significant difference between classes. SGLT2i show a numerically larger HFH reduction, but the difference is not statistically robust. Evidence is largely derived from remote MI, limiting its applicability to the acute post-infarction setting. Treatment choice should be individualized based on patient risk profiles. Dedicated head-to-head trials are urgently needed.

**Systematic review registration:**

https://www.crd.york.ac.uk/PROSPERO/view/CRD420261299756, identifier CRD420261299756.

## 1 Background

Type 2 diabetes mellitus (T2DM) and atherosclerotic cardiovascular disease (ASCVD), particularly a history of myocardial infarction (MI), frequently coexist, creating a synergistically high-risk population with substantial morbidity and mortality (1). Patients with T2DM who have experienced a prior MI face a markedly elevated risk of recurrent cardiovascular events (2), heart failure, and cardiovascular death compared to their non-diabetic counterparts (3). This heightened risk persists despite standard secondary prevention therapies, including antiplatelet agents, statins, and renin-angiotensin-aldosterone system inhibitors, underscoring a critical unmet need for additional cardioprotective strategies in this vulnerable group (4).

The landscape of T2DM management has been transformed by the advent of two novel drug classes: sodium-glucose cotransporter-2 (SGLT2) inhibitors and glucagon-like peptide-1 receptor agonists (GLP-1 RAs) (5). Originally developed for glycemic control, large-scale cardiovascular outcome trials (CVOTs) have unequivocally demonstrated their significant benefits in reducing major adverse cardiovascular events (MACE) in patients with T2DM and established ASCVD or high cardiovascular risk (6, 7). SGLT2 inhibitors exhibit a pronounced benefit in reducing hospitalization for heart failure (HFH) and progression of renal disease; conversely, GLP-1 RAs show robust reductions in atherosclerotic events (7–10).

While the cardioprotective effects of both classes are established in broad CVOT populations, a crucial clinical question remains regarding their relative efficacy specifically in patients with a history of MI—a group at particularly high risk for recurrent events. However, it is critical to note upfront that, to date, no head-to-head randomized controlled trials (RCTs) have directly compared SGLT2 inhibitors and GLP-1 receptor agonists in this or any other population. Consequently, the present analysis relies on an indirect comparison of separate placebo-controlled trials (10). Furthermore, the majority of existing CVOTs that reported data for patients with a prior MI enrolled individuals with a median time since their last MI of several years (e.g., a median of 5.4 years in the DECLARE-TIMI 58 trial) (11). This evidence base does not ideally represent the clinical scenario of a “recent” MI (typically defined as within 90–180 days), which represents a period of particularly heightened vulnerability. These fundamental limitations—the indirect nature of the comparison and the predominance of remote rather than recent MI populations (12, 13) —govern the interpretation of all findings presented in this meta-analysis.

Therefore, the primary objective of this study was not to claim a direct comparative superiority, but rather to synthesize the available evidence from RCTs to indirectly compare the cardiovascular effects of SGLT2 inhibitors and GLP-1 receptor agonists in patients with T2DM and a history of MI, with a secondary exploratory focus on the limited subset of data available for patients with a more recent MI. We hypothesized that both classes would confer significant cardiovascular benefit compared to placebo/standard care, but that their effect profiles might differ, with SGLT2 inhibitors showing a greater relative risk reduction for HFH and GLP-1 RAs showing greater benefit for atherosclerotic MACE components. All comparative findings are presented as exploratory and hypothesis-generating.

## 2 Methods

### 2.1 Study design and registration

This systematic review and meta-analysis was conducted according to the Cochrane Handbook for Systematic Reviews of Interventions (Version 6.4, 2023) (14) and is reported following the Preferred Reporting Items for Systematic Reviews and Meta-Analyses (PRISMA) 2020 statement (15). A completed PRISMA 2020 checklist is provided in **Supplementary File 1**. The study protocol was registered *a priori* on the International Prospective Register of Systematic Reviews (PROSPERO) (Registration ID: CRD420261299756).

### 2.2 Eligibility criteria

Studies were selected based on the PICOS framework (16):

Population: Adult patients (≥18 years) with T2DM and a history of myocardial infarction (MI). The primary analysis included all participants with prior MI, regardless of time since the event. A secondary exploratory subgroup analysis was performed for “recent MI” (≤180 days from randomization) versus “chronic MI” (>180 days), where data permitted. This refinement acknowledges that the majority of available trials enrolled patients with a remote MI (median time >5 years in DECLARE-TIMI 58), and only the EMPACT-MI and ELIXA trials specifically targeted acute coronary syndrome populations.

Intervention: SGLT2 inhibitors (canagliflozin, dapagliflozin, empagliflozin, ertugliflozin, sotagliflozin) or GLP-1 receptor agonists (liraglutide, semaglutide, dulaglutide, exenatide, lixisenatide, albiglutide, tirzepatide – though no CVOT data available for tirzepatide at the time of search) at any approved dose.

Comparator: Placebo or standard of care (with or without background glucose-lowering therapy).

Outcomes: Primary Outcome: Major Adverse Cardiovascular Events (MACE), defined as a composite of cardiovascular death, non-fatal MI, or non-fatal stroke.

Secondary outcomes: Individual components of MACE (cardiovascular death, myocardial infarction, stroke), hospitalization for heart failure (HHF/HFH), and all-cause mortality.

Safety Outcomes: We pre-specified the following safety outcomes: incident severe hypoglycemia, diabetic ketoacidosis (for SGLT2i), pancreatitis, and any serious adverse events (SAEs) as reported. However, due to anticipated inconsistent reporting across trials, we planned a narrative summary rather than a quantitative meta-analysis for these outcomes.

Study design: Phase III randomized controlled trials (RCTs) with a minimum follow-up of 12 months.

Exclusion criteria included: non-randomized studies, trials without a placebo/active control arm, trials in patients with type 1 diabetes or without T2DM, trials where the intervention was initiated in the acute inpatient setting (<72 hours post-MI) unless specified as a dedicated post-ACS trial, and duplicate publications.

### 2.3 Information sources and search strategy

A systematic and comprehensive literature search was performed from database inception to February 1, 2026 across the following electronic databases: PubMed/MEDLINE, Embase, Cochrane Central Register of Controlled Trials (CENTRAL), and ClinicalTrials.gov. No language restrictions were applied initially. The search strategy was developed in consultation with a medical librarian and combined controlled vocabulary (MeSH, Emtree) and free-text terms related to: 1) T2DM, 2) Myocardial Infarction/Acute Coronary Syndrome, 3) SGLT2 inhibitors and GLP-1 receptor agonists (including all individual drug names), and 4) Randomized Controlled Trials. A highly sensitive RCT filter was applied where appropriate. The detailed search strategy for PubMed is provided in the supplementary materials (**Supplementary Table 2**). The reference lists of all included studies and relevant review articles were manually scanned to identify any additional eligible trials.

### 2.4 Study selection and data collection process

Search results were imported into the Rayyan web application for systematic reviews. After removal of duplicates, two independent reviewers (YX R and H Y) screened titles and abstracts against the eligibility criteria. Full texts of potentially relevant articles were then retrieved and assessed independently by the same reviewers. Any discrepancies at any stage were resolved through discussion or by consultation with a third senior reviewer (S Z). The study selection process was documented using a PRISMA flow diagram.

A pre-piloted, standardized data extraction form was used. Two reviewers (TX H and QY T) independently extracted the following data from each included study: first author, year of publication, trial name, clinical trial registration number, study design, participant characteristics (sample size, mean age, diabetes duration, time since most recent MI, baseline HbA1c, renal function, left ventricular ejection fraction), intervention and comparator details (drug, dose, frequency), duration of follow-up, and outcome data (number of events for each arm). For time-to-event outcomes, hazard ratios (HRs) with 95% confidence intervals (CIs) were extracted preferentially. If not reported, effect estimates were calculated from available event numbers and patient-years, or digitized from Kaplan-Meier curves using validated software (WebPlotDigitizer) (17, 18). Corresponding authors were contacted via email to request missing or unclear data.

### 2.5 Risk of bias and quality assessment

The risk of bias for each individual RCT was assessed independently by two reviewers using the revised Cochrane Risk of Bias tool for randomized trials (RoB 2.0) (19, 20), focusing on five domains: randomization process, deviations from intended interventions, missing outcome data, measurement of the outcome, and selection of the reported result. Judgments were categorized as “low risk,” “some concerns,” or “high risk.” Disagreements were resolved by consensus.

The overall certainty of evidence for each meta-analyzed outcome was evaluated using the Grading of Recommendations, Assessment, Development, and Evaluations (GRADE) framework (21, 22). The evidence from RCTs started as high quality and was downgraded based on assessments of risk of bias, inconsistency, indirectness, imprecision, and publication bias. We pre-specified that downgrading for indirectness would be applied if most included studies did not exclusively enroll patients with recent MI (≤180 days). Downgrading for imprecision would be applied if the 95% confidence interval crossed the line of no effect or if the total number of events was small (<300).

### 2.6 Data synthesis and statistical analysis

All statistical analyses were performed using R statistical software (version 4.3.1) with the ‘meta’ and ‘metafor’ packages. For dichotomous outcomes, pooled hazard ratios (HRs) with 95% CIs were calculated using a generic inverse-variance method under a random-effects model, which incorporates both within-study and between-study variance. The DerSimonian-Laird estimator was used for tau². Statistical heterogeneity was assessed using the Cochran’s Q test (significance level p < 0.10) and quantified using the I² statistic, with values of 25%, 50%, and 75% representing low, moderate, and high heterogeneity, respectively (23, 24). In addition, we calculated the 95% confidence intervals for I² using the ‘hetplot’ function in the ‘meta’ package, acknowledging that these intervals are wide when the number of studies is small.

To address the absence of head-to-head RCTs comparing SGLT2i and GLP-1 RAs, we supplemented the original Bucher interaction-test approach with a formal frequentist network meta-analysis (NMA), which was used as the primary comparative analysis. The NMA was conducted using the graph-theoretical method implemented in the R package netmeta (version 3.2-0) under a random-effects model with the DerSimonian-Laird estimator for τ². This approach simultaneously synthesizes direct (drug vs. placebo) and indirect evidence in a single statistical model, yielding relative effect estimates for every treatment comparison and allowing for the computation of P-scores (frequentist analogues of SUCRA) to rank treatments. We chose the NMA because it formally borrows strength from the entire evidence network, provides more precise indirect comparisons than the simple Bucher method, and is the current standard for indirect treatment comparisons in systematic reviews. Because the network was star-shaped (all studies compared a single active drug to placebo, with no closed loops), inconsistency between direct and indirect evidence could not be assessed.

The original Bucher interaction test, which compares class-specific pooled HRs from separate placebo-controlled trials via subgroup interaction, was retained as a pre-specified sensitivity analysis to verify the robustness of the NMA results.

To avoid double-counting from overlapping study populations, we applied the following rules: (1)For the EMPACT-MI trial, we used the overall T2DM population results as the primary source and did not include the separate “EMPACT-MI T2DM Subgroup” as an independent entry in the main analysis, because the subgroup patients were a subset of the overall trial population; (2) For the REWIND trial, we used the reported CVD subgroup data (patients with prior MI or revascularization) and did not additionally include the overall REWIND population to prevent overlap; (3) A sensitivity analysis was performed excluding all pre-specified subgroup-derived data (e.g., DECLARE-TIMI 58 MI subgroup, REWIND CVD subgroup) to test the robustness of the primary findings against potential double-counting.

Pre-specified subgroup analyses were conducted to explore potential sources of heterogeneity and compare effect estimates between: 1) Drug class (SGLT2 inhibitors vs. GLP-1 RAs); 2) Timing of MI relative to randomization (recent MI: median time from MI to randomization ≤180 days vs. chronic MI: >180 days, where data permitted); and 3) Route of administration for GLP-1 RAs (subcutaneous vs. oral). Interaction tests were performed to assess for statistically significant differences between subgroups.

Because no head-to-head RCTs exist, all comparisons between SGLT2i and GLP-1 RAs are indirect. We used interaction tests on class-specific pooled estimates from separate placebo-controlled trials. This approach does not constitute a formal network meta-analysis, and results are presented as exploratory.

In the NMA, for each outcome we produced: a network graph illustrating the evidence geometry; forest plots showing the random-effects NMA estimates of each treatment versus placebo; league tables providing the HR and 95% CI for all possible pairwise treatment comparisons; and P-score bar charts to rank the treatments by efficacy (where a P-score close to 1 indicates the best treatment).

Sensitivity analyses were performed to test the robustness of the primary findings, including: 1) Leave-one-out analysis for the primary outcome (MACE); 2) Using a fixed-effect model; and 3) Excluding studies judged to have “some concerns” or “high risk” of bias.

Publication bias was assessed visually using funnel plots for outcomes with at least 10 studies. For outcomes with fewer than 10 studies, we did not perform formal Egger’s regression test because it is known to be underpowered and potentially misleading (24). A two-sided p-value <0.05 was considered statistically significant for all analyses except where otherwise noted for heterogeneity and publication bias tests.

### 2.7 Safety outcomes synthesis

Given the anticipated heterogeneity in safety outcome definitions and reporting across trials, we did not pool safety data quantitatively. Instead, we extracted available safety outcome data (severe hypoglycemia, DKA, pancreatitis, SAEs) from each included trial and present a narrative summary in the supplementary materials (**Supplementary Table 2**). This approach allows us to report available safety information without producing potentially misleading pooled estimates from inconsistent data.

### 2.8 Indirect comparison and handling of limitations

This meta-analysis does not include any head-to-head RCTs directly comparing SGLT2 inhibitors versus GLP-1 receptor agonists. All effect estimates for each drug class are derived from separate placebo-controlled trials. The comparison between classes is made via interaction tests on class-pooled estimates. This indirect comparison is subject to confounding by differences in baseline patient characteristics, trial designs, and follow-up durations across trials that are not matched. Therefore, all p-values for interaction and statements about comparative efficacy should be interpreted as hypothesis-generating rather than conclusive. We have explicitly acknowledged this limitation in the title, abstract, and discussion.

## 3 Results

### 3.1 Study selection and characteristics

**Figure 1:**
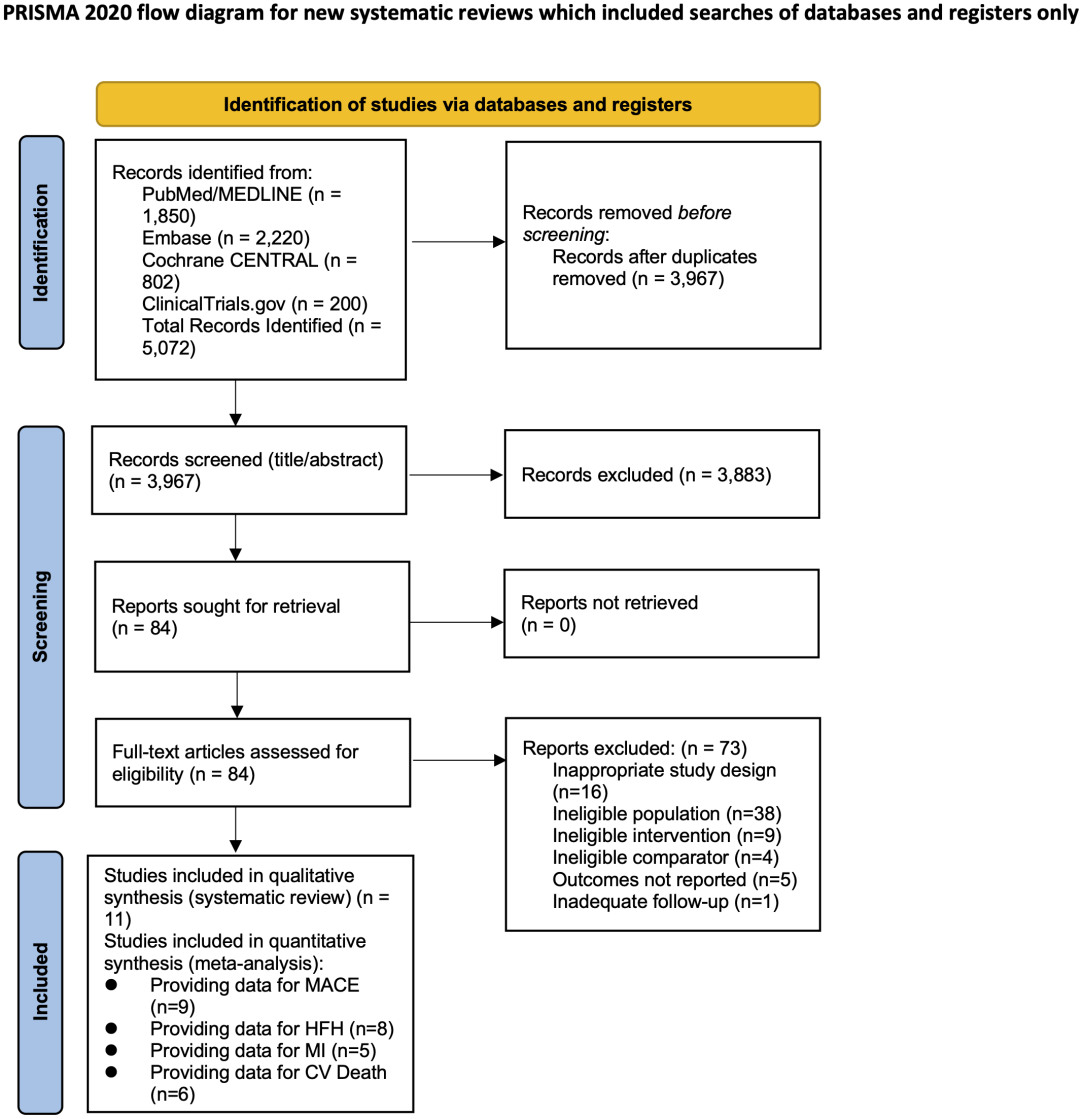
PRISMA flow diagram of the study selection process. Flow diagram illustrating the identification, screening, eligibility assessment, and inclusion of studies in this systematic review and network meta-analysis, in accordance with PRISMA 2020 guidelines. Searches of PubMed/MEDLINE, Embase, Cochrane CENTRAL, and ClinicalTrials.gov (inception to February 1, 2026) yielded 5,072 records. After removal of duplicates, 3,967 records were screened and 84 full-text articles assessed. Eleven unique RCTs met the inclusion criteria and were included in the systematic review. Following exclusion of overlapping populations, the final quantitative syntheses comprised 7 studies for MACE, 6 for HFH, 4 for MI, and 6 for CV death.

The systematic literature search and selection process are detailed in the PRISMA flow diagram (**Figure 1**). A total of 5,072 records were identified through database searching from inception to February 1, 2026: PubMed/MEDLINE (n = 1,850), Embase (n = 2,220), Cochrane CENTRAL (n = 802), and ClinicalTrials.gov (n = 200). After removing 1,105 duplicates, 3,967 unique records underwent title and abstract screening. Of these, 3,883 records were excluded as clearly irrelevant. The remaining 84 full-text articles were assessed for eligibility against the pre-defined PICOS criteria.

During full-text review, 73 articles were excluded for the following reasons: non-randomized study design (n=16), population not restricted to or not reporting separate data for patients with type 2 diabetes and myocardial infarction (n=38), intervention not being an SGLT2 inhibitor or GLP-1 receptor agonist (n=9), comparator not being placebo/standard care (n=4), outcome of interest not reported (n=5), and follow-up duration less than 12 months (n=1).

Ultimately, 11 unique randomized controlled trials (RCTs) met all inclusion criteria and were included in the systematic review (25–35). However, to avoid double-counting from overlapping populations (as detailed in Section 2.6), we applied the following adjustments: (1) For EMPACT-MI, we used the overall T2DM population results (34) and excluded the separate “EMPACT-MI T2DM Subgroup” entry (30) from the main analysis; (2) For REWIND, we used the reported CVD subgroup data (patients with prior MI or revascularization) (26) and did not additionally include the overall REWIND population. After applying these adjustments, the final numbers of studies contributing to each meta-analysis endpoint were as follows: (1) Major Adverse Cardiovascular Events (MACE): 7 studies (2 SGLT2i, 5 GLP-1 RA) (originally 9; excluded EMPACT-MI T2DM subgroup and the overall REWIND population, retaining only the REWIND CVD subgroup); (2) Heart Failure Hospitalization (HFH): 6 studies (2 SGLT2i, 4 GLP-1 RA) (originally 8; same exclusions); (3) Myocardial Infarction (MI): 4 studies (1 SGLT2i, 3 GLP-1 RA) (originally 5; excluded REWIND overall); (4) Cardiovascular Death (CV Death): 6 studies (2 SGLT2i, 4 GLP-1 RA) (originally 7; excluded REWIND overall and the EMPACT-MI T2DM subgroup). Detailed characteristics of the 11 included trials are summarized in **Supplementary Table 3**.

### 3.2 GRADE evidence quality assessment

We assessed the quality of evidence for each outcome using the Grading of Recommendations, Assessment, Development and Evaluations (GRADE) framework. The initial quality for all outcomes was deemed High, as they were derived from randomized controlled trials. The final rating for each outcome was then determined by evaluating five domains: risk of bias, inconsistency, indirectness, imprecision, and publication bias. Downgrading occurred if significant concerns were identified in any domain.

The detailed GRADE Summary of Findings table (**Table 1**) presents the certainty of evidence separately for each comparison (SGLT2i vs. Placebo, GLP-1 RA vs. Placebo, and the indirect comparison SGLT2i vs. GLP-1 RA), with absolute effect estimates.

**Table 1:** GRADE summary of findings for cardiovascular outcomes comparing SGLT2 inhibitors, GLP-1 receptor agonists, and their indirect comparison in patients with type 2 diabetes and a history of myocardial infarction.

Outcome	Comparison	No of studies (participants)	Risk of bias	Inconsistency	Indirectness	Imprecision	Publication bias	Relative effect HR (95% CI)	Anticipated absolute effects (per 1000 patients)	Quality	Rationale
MACE	SGLT2i vs Placebo	2 (8509)	Not serious	Not serious	Serious¹	Not serious	None	0.87 (0.77–0.97)	Placebo: 116 SGLT2i: 101 Difference: 15 fewer	⊕⊕⊕◯ Moderate	¹Population mostly had remote MI history (median >5 years in DECLARE-TIMI 58); only EMPACT-MI enrolled recent MI.
	GLP-1 RA vs Placebo	5 (17,254)	Not serious	Not serious	Serious¹	Not serious	None	0.83 (0.77–0.89)	Placebo: 116 GLP-1 RA: 96 Difference: 20 fewer	⊕⊕⊕◯ Moderate	¹Same indirectness concern as above.
	SGLT2i vs GLP-1 RA (indirect)	7 (25,763)	Not serious	Not serious	Serious¹˙²	Serious³	None	1.05 (0.92–1.20)	—	⊕⊕◯◯ Low	¹Predominantly remote MI populations; ²Indirect comparison; ³CI includes 1.0, limited data for indirect estimate.
HFH	SGLT2i vs Placebo	2 (8509)	Not serious	Not serious	Serious^4^	Not serious	None	0.74 (0.62–0.90)	Placebo: 56 SGLT2i: 41 Difference: 15 fewer	⊕⊕⊕◯ Moderate	^4^Evidence comes from trials with mixed MI timing; only EMPACT-MI enrolled recent MI directly.
	GLP-1 RA vs Placebo	4 (13,733)	Not serious	Not serious	Serious^4^	Not serious	None	0.90 (0.81–1.00)	Placebo: 56 GLP-1 RA: 50 Difference: 6 fewer	⊕⊕⊕◯ Moderate	^4^Same indirectness as above.
	SGLT2i vs GLP-1 RA (indirect)	6 (22,242)	Not serious	Not serious	Serious^4^˙^5^	Serious³	None	0.83 (0.67–1.03)	—	⊕⊕◯◯ Low	^4^Mixed MI populations; ^5^Indirect comparison; ³CI includes 1.0, imprecision.
MI	SGLT2i vs Placebo	1 (3584)	Not serious	Not serious	Serious^6^;	Very serious^7^	None	0.78 (0.64–0.96)	Placebo: 59 SGLT2i: 46 Difference: 13 fewer	⊕◯◯◯ Very low	^6^;Single study of remote MI (DECLARE-TIMI 58); ^7^Only one trial, cannot assess consistency.
	GLP-1 RA vs Placebo	3 (8975)	Not serious	Not serious	Serious^6^;	Not serious	None	0.74 (0.66–0.85)	Placebo: 59 GLP-1 RA: 44 Difference: 15 fewer	⊕⊕⊕◯ Moderate	^6^;Same indirectness as above.
	SGLT2i vs GLP-1 RA (indirect)	4 (12,559)	Not serious	Not serious	Serious^6^;˙^8^	Very serious^9^	None	1.05 (0.83–1.33)	—	⊕◯◯◯ Very low	^6^;Mostly remote MI; ^8^Indirect comparison; ^9^Very few studies, wide CI.
CV Death	SGLT2i vs Placebo	2 (10,106)	Not serious	Not serious	Serious¹^0^	Serious¹¹	None	0.98 (0.82–1.18)	Placebo: 41 SGLT2i: 40 Difference: 1 fewer	⊕⊕◯◯ Low	¹^0^Evidence from EMPACT-MI (recent MI) and DECLARE-TIMI 58 MI subgroup (remote MI); ¹¹Only two trials, CI includes both benefit and harm.
	GLP-1 RA vs Placebo	4 (25,593)	Not serious	Not serious	Serious¹^0^	Serious¹²	None	0.92 (0.83–1.02)	Placebo: 41 GLP-1 RA: 38 Difference: 3 fewer	⊕⊕◯◯ Low	¹^0^Mixed MI timing; ¹²CI crosses 1.0.
	SGLT2i vs GLP-1 RA (indirect)	6 (35,699)	Not serious	Not serious	Serious¹^0^˙¹³	Serious^9^	None	0.94 (0.76–1.16)	—	⊕⊕◯◯ Low	¹^0^Mixed populations; ¹³Indirect comparison; ^9^Wide CI includes both benefit and harm.

MACE, major adverse cardiovascular events; HFH, hospitalization for heart failure; MI, myocardial infarction; CV Death, cardiovascular death; HR, hazard ratio; CI, confidence interval; SGLT2i, sodium-glucose cotransporter-2 inhibitor; GLP-1 RA, glucagon-like peptide-1 receptor agonist.

• Absolute effects were approximated from pooled placebo arm event rates across included trials (MACE 116/1000, HFH 56/1000, MI 59/1000, CV Death 41/1000) assuming a median follow-up of 2–3 years.

• ¹, ^4^, ^6^, ¹^0^ Indirectness: The majority of evidence derives from trials enrolling patients with a *history* of MI (often remote, e.g., DECLARE-TIMI 58 median 5.4 years) rather than exclusively recent MI (≤180 days).

• ², ^5^, ^8^, ¹³ Indirectness due to indirect comparison design (no head-to-head RCTs).

• ³, ^9^ Imprecision: Confidence interval includes the possibility of both important benefit and harm; indirect comparison included few trials.

This table presents the Grading of Recommendations Assessment, Development and Evaluation (GRADE) certainty of evidence for each outcome and each comparison separately. Columns show the outcome, the specific comparison (SGLT2i vs. Placebo, GLP-1 RA vs. Placebo, or the indirect comparison SGLT2i vs. GLP-1 RA), the number of studies and total participants, and the ratings for five domains: risk of bias, inconsistency, indirectness, imprecision, and publication bias. The relative effect is expressed as hazard ratio (HR) with 95% confidence interval (CI) from the random-effects network meta-analysis. Anticipated absolute effects are presented as events per 1,000 patients treated, calculated by multiplying the pooled placebo-arm event rate (derived from included trials: MACE 116/1,000, HFH 56/1,000, MI 59/1,000, CV death 41/1,000, assuming a median follow-up of approximately 2–3 years) by the corresponding HR. The overall quality of evidence (rightmost column) is rated as High (⊕⊕⊕⊕), Moderate (⊕⊕⊕◯), Low (⊕⊕◯◯), or Very low (⊕◯◯◯). Downgrading reasons are numbered in the Rationale column and explained in the table footnote: ¹, ^4^, ^6^, ¹^0^ Indirectness because the majority of evidence derived from trials enrolling patients with a history of MI, often remote (e.g., DECLARE-TIMI 58 median 5.4 years since MI), rather than exclusively recent MI (≤180 days); ², ^5^, ^8^, ¹³ Indirectness arising from the indirect comparison design (no head-to-head RCTs); ³, ^9^ Imprecision because the 95% CI includes the possibility of both clinically important benefit and harm; ^7^, ¹¹ Very serious imprecision as only a single study contributed data for the SGLT2i class, precluding assessment of consistency. MACE, major adverse cardiovascular events; HFH, hospitalization for heart failure; MI, myocardial infarction; CV death, cardiovascular death.

The overall quality of evidence was Moderate for MACE and HFH (for both drug-class versus placebo comparisons), and Low to Very low for MI and CV death, as well as for all indirect comparisons between drug classes.

All included studies were well-conducted, multicenter, double-blind, placebo-controlled RCTs with low risk of bias across key domains such as randomization, allocation concealment, and blinding. Therefore, no downgrading was applied for risk of bias. Consistency (inconsistency) was exceptional across all analyses, with I² statistics consistently at 0% or near 0%, and overlapping confidence intervals, indicating homogeneous treatment effects; thus, no downgrading was applied for inconsistency. Visual inspection of funnel plots revealed no significant evidence of publication bias for any endpoint (formal Egger’s test was not performed due to fewer than 10 studies per outcome), so no downgrading was applied on this basis.

However, indirectness was a key factor leading to downgrading for most outcomes. The majority of included trials, while containing patients with prior myocardial infarction (MI), were not exclusively designed for or limited to patients with recent MI (typically defined as within 90–180 days). For instance, trials such as DECLARE-TIMI 58 (Dapagliflozin), Harmony Outcomes (Albiglutide), SUSTAIN-6 (Semaglutide), PIONEER 6 (Oral semaglutide), SOUL (Oral semaglutide), and the REWIND CVD subgroup (Dulaglutide) enrolled broad populations of patients with T2DM and either established atherosclerotic cardiovascular disease (a category that includes prior MI) or multiple risk factors. The baseline time from the most recent MI in these studies was often several years (e.g., a median of 5.4 years in DECLARE-TIMI 58 MI subgroup), which does not perfectly align with the “recent MI” focus of our review. Only the EMPACT-MI trial (Empagliflozin) specifically targeted patients with a recent acute MI (within 14 days), and the ELIXA trial (Lixisenatide) enrolled patients within 180 days of an acute coronary syndrome (though it did not contribute to the quantitative synthesis). Consequently, the evidence was downgraded by one level for indirectness in all direct comparisons. For the indirect NMA comparisons between SGLT2i and GLP-1 RA, an additional level of indirectness was applied owing to the absence of head-to-head trials.

Imprecision was another contributing factor, particularly for the MI and CV death outcomes. The meta-analysis for MI included only 4 studies (1 SGLT2i, 3 GLP-1 RA), and for CV death, 6 studies (2 SGLT2i, 4 GLP-1 RA). The confidence interval for the NMA-based indirect comparison for CV death (0.76–1.16) was wide and crossed the line of no effect, indicating substantial statistical uncertainty. Similarly, the indirect comparison for MI (HR 1.05, 95% CI 0.83–1.33) also included both potential benefit and harm. For direct class-versus-placebo comparisons, MI for SGLT2i was informed by a single study and judged as very serious imprecision; CV death for GLP-1 RA crossed 1.0 and was downgraded for imprecision. Therefore, the evidence for MI and CV death was downgraded further for imprecision, resulting in a Low or Very low rating for these outcomes.

**Figure 2:**
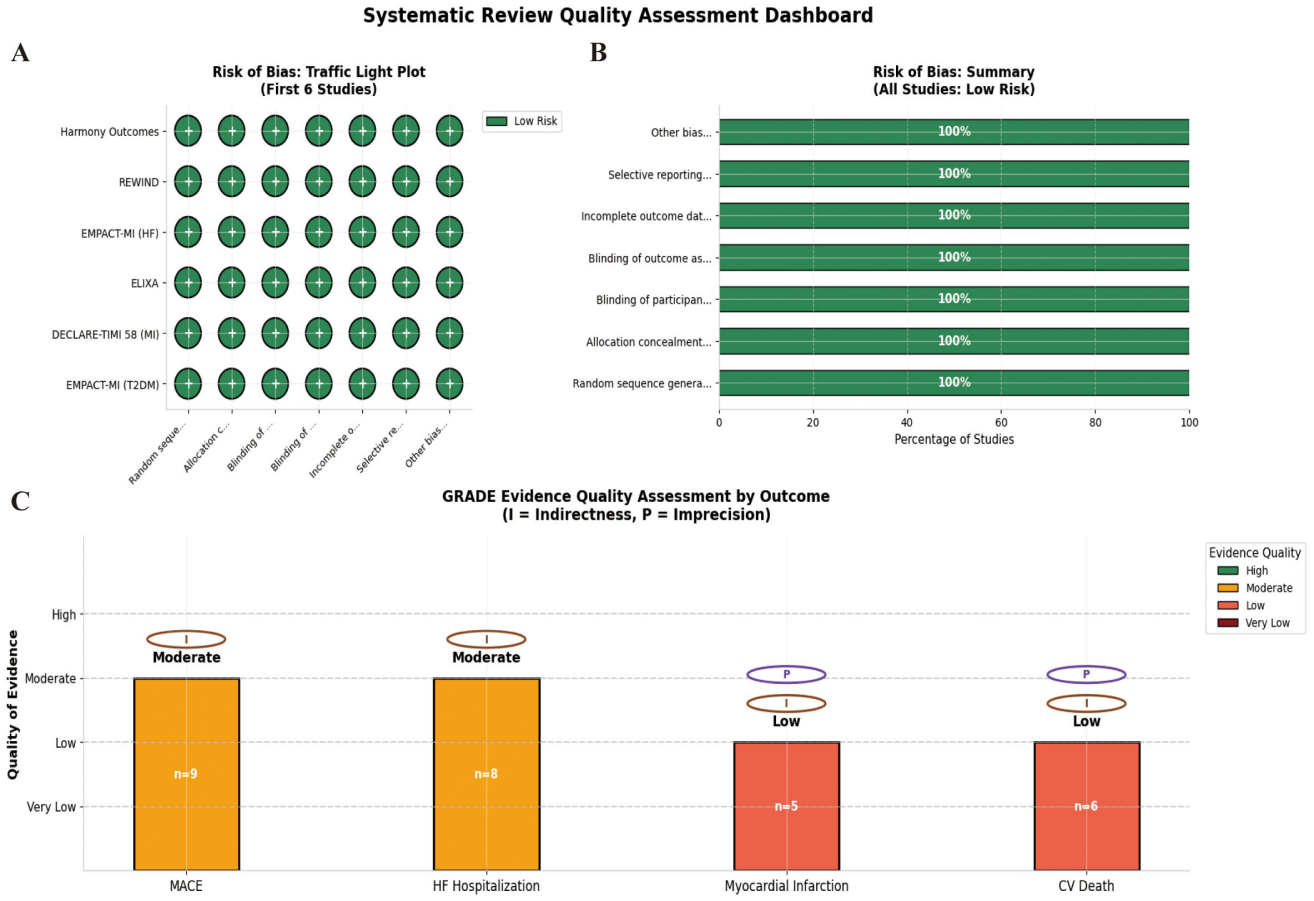
Systematic review quality assessment dashboard. **(A)** Risk of bias traffic light plot for representative studies using the Cochrane RoB 2 tool; green indicates low risk of bias. All 11 included RCTs were judged at low risk of bias across all domains. **(B)** Summary bar chart of risk of bias across domains. **(C)** GRADE evidence profile summarizing the final certainty ratings for MACE, HFH, MI, and CV death. Downgrading factors are indicated by the letters I (indirectness) and P (imprecision).

No factors were identified that would warrant upgrading the quality of evidence (e.g., large magnitude of effect or dose-response gradient). In summary, while the underlying data are robust and consistent, the strength of our conclusions is moderated by the limited directness of the evidence to the specific “recent MI” population, the inherent limitations of indirect comparisons, and, for some endpoints, limited precision. The GRADE evidence profile for each outcome is presented in **Table 1** and summarized graphically in **Figure 2**.

### 3.3 Primary outcome: major adverse cardiovascular events

#### 3.3.1 Combined class meta-analysis

The original combined-class random-effects meta-analysis, which pooled all seven included studies regardless of drug class versus placebo, showed a statistically significant reduction in MACE risk (pooled HR 0.838, 95% CI 0.789–0.890; p < 0.001) (**Supplementary Figure 1**). Heterogeneity was negligible (I² = 0.0%, 95% CI 0.0%–70.8%; τ² = 0.0000; Cochran’s Q p = 0.779), indicating highly consistent treatment effects across the included trials. When the analysis was stratified by drug class using the Bucher interaction test, the class-specific pooled HRs were 0.88 (95% CI 0.79–0.98) for SGLT2i and 0.84 (95% CI 0.79–0.89) for GLP-1 RAs (**Supplementary Figure 2**). The interaction test yielded a non-significant p-value of 0.48. However, this test does not formally borrow strength across the entire network and is thus considered a sensitivity analysis.

#### 3.3.2 Network meta-analysis (primary comparative analysis)

**Figure 3:**
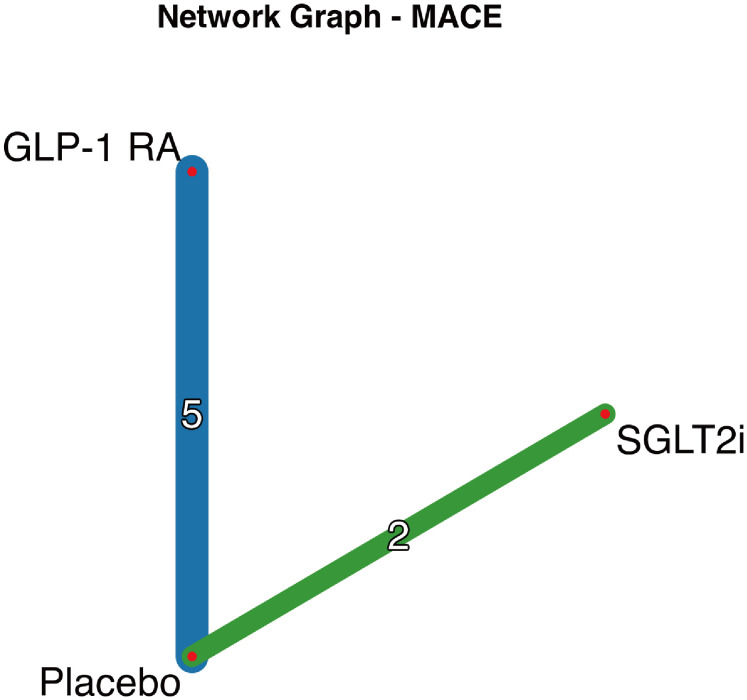
Network graph for major adverse cardiovascular events (MACE). Star-shaped evidence network for MACE (7 studies: 2 SGLT2i, 5 GLP-1 RA). Nodes represent treatments (Placebo, SGLT2i, GLP-1 RA); edges represent direct pairwise comparisons. All comparisons are against Placebo; no head-to-head trials exist.

**Figure 4:**
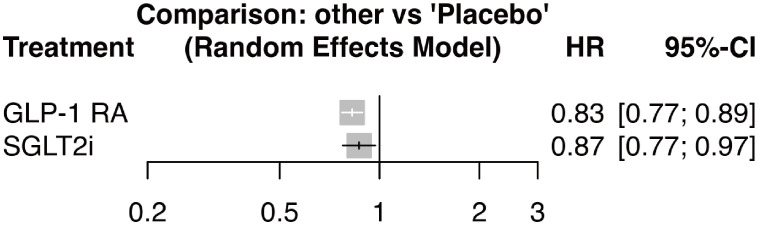
Network meta-analysis forest plot for major adverse cardiovascular events (MACE). Random-effects NMA estimates of each treatment compared with Placebo. SGLT2i: HR 0.87 (95% CI 0.77–0.97); GLP-1 RA: HR 0.83 (95% CI 0.77–0.89). Heterogeneity: τ² = 0, I² = 0%.

To obtain a more rigorous indirect comparison, a frequentist network meta-analysis (NMA) was conducted using the same seven trials. The network geometry was star-shaped, with Placebo serving as the central node and both SGLT2i and GLP-1 RA connected only to Placebo, reflecting the absence of any direct head-to-head comparisons (**Figure 3**). Under the random-effects model, both drug classes significantly reduced MACE risk relative to placebo. The NMA estimate for GLP-1 RAs was HR 0.83 (95% CI 0.77–0.89), and for SGLT2i was HR 0.87 (95% CI 0.77–0.97) (**Figure 4**). Between-study variance was estimated as τ² = 0, and I² = 0%, confirming the homogeneity observed in the pairwise meta-analyses.

**Figure 5:**
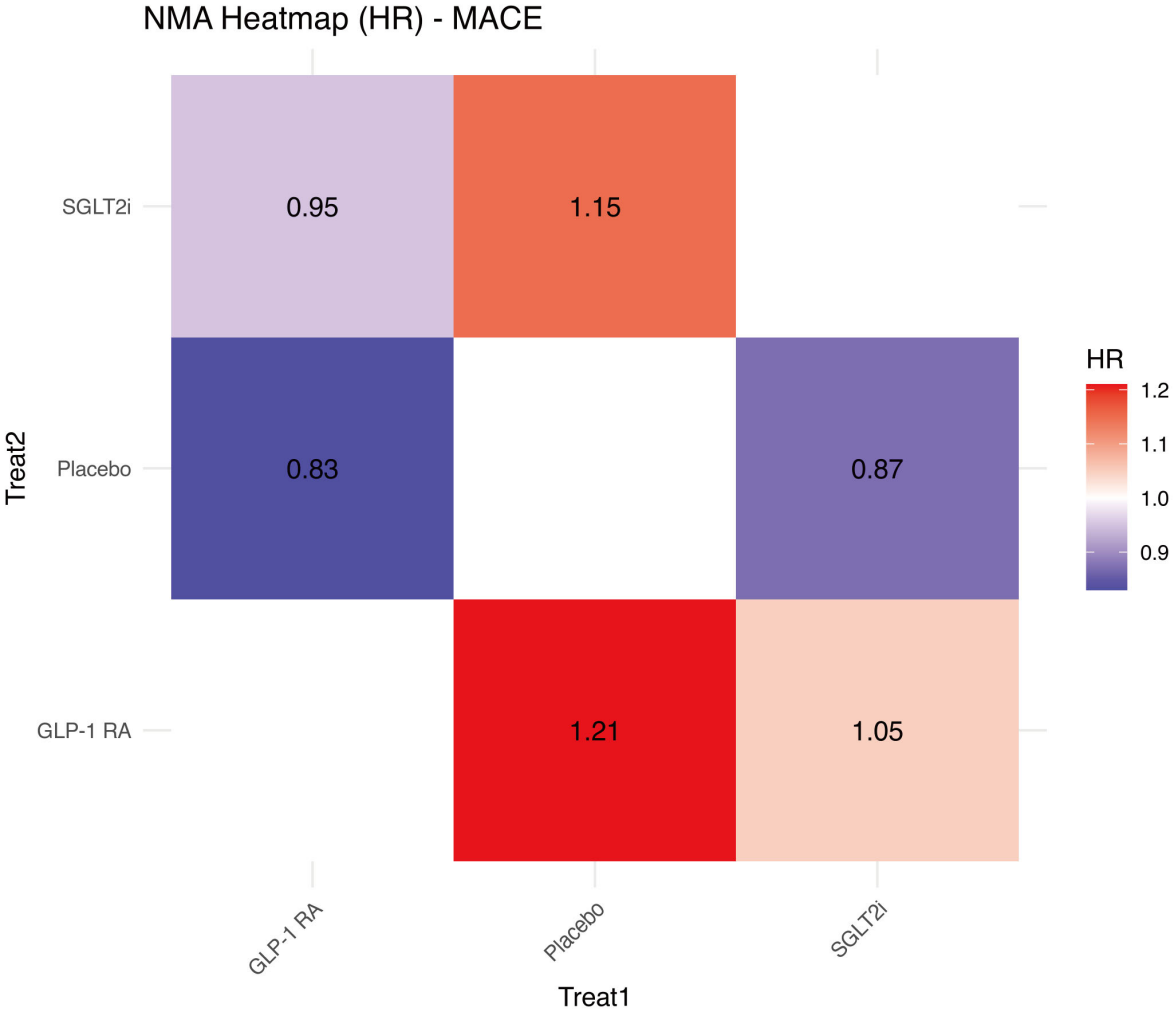
League table heatmap for major adverse cardiovascular events (MACE). Heatmap representation of the NMA league table displaying pairwise treatment comparisons as hazard ratios. Colour scale: blue (HR < 1, protective) to red (HR > 1, harmful). Diagonal entries (treatment vs. itself) are left blank. Numerical HR values are overlaid on the tiles.

**Figure 6:**
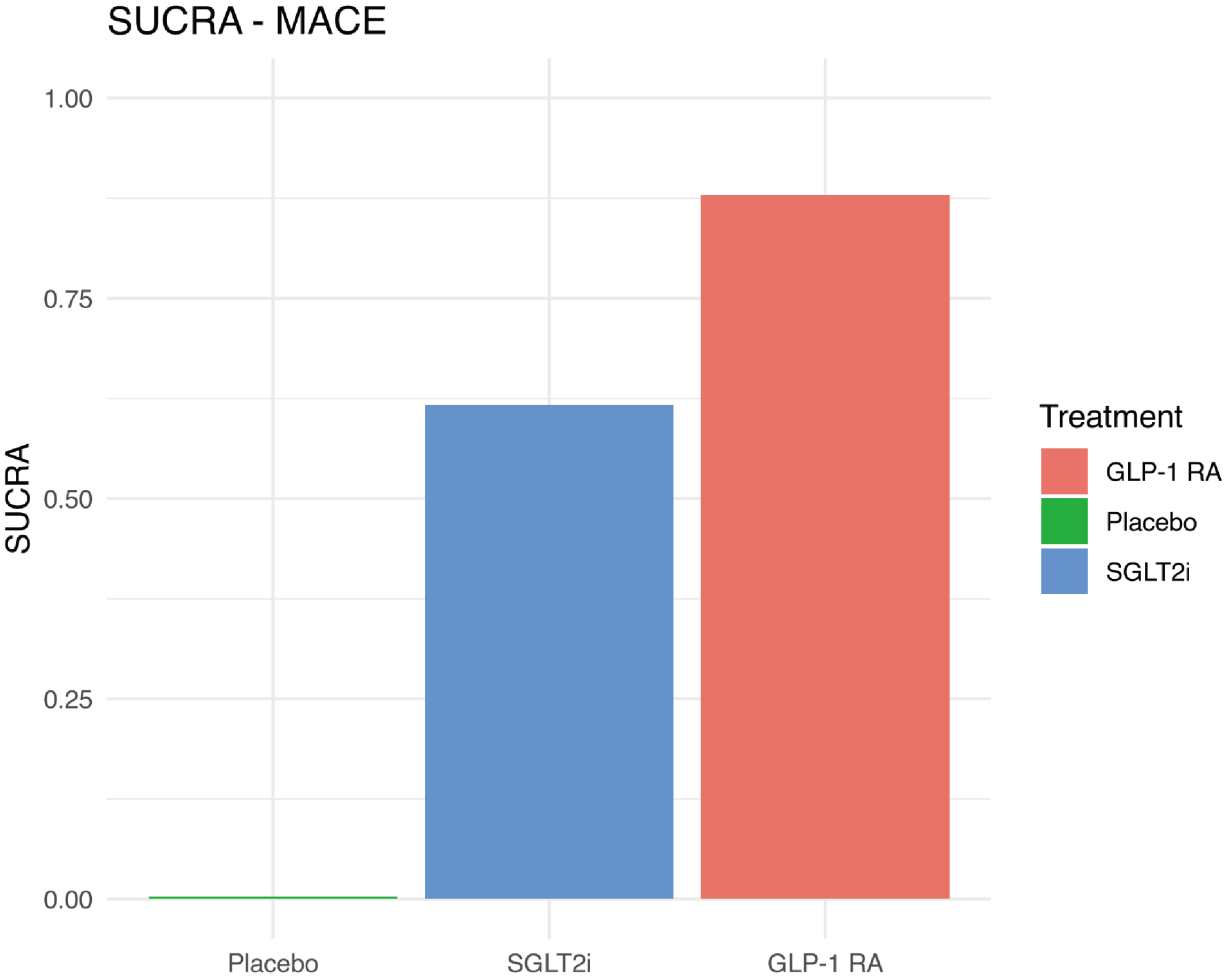
P-score ranking for major adverse cardiovascular events (MACE). Bar chart of P-scores (frequentist analogues of SUCRA). Higher scores indicate greater probability of being the best treatment. GLP-1 RAs: 0.89; SGLT2i: 0.57; Placebo: 0.04.

The primary interest lay in the indirect comparison between SGLT2i and GLP-1 RAs. From the NMA, the estimated HR for SGLT2i versus GLP-1 RAs was 1.05 with a 95% confidence interval spanning 0.92 to 1.20, and a random-effects NMA p-value of 0.44, indicating that no statistically significant difference could be detected between the two classes. The complete set of pairwise treatment comparisons is displayed in the league table heatmap (**Figure 5**), where the colour gradient (blue for HR < 1, red for HR > 1) provides a visual summary of the relative effects. To further facilitate the interpretation of comparative efficacy, we computed P-scores, the frequentist equivalent of the surface under the cumulative ranking curve (SUCRA). GLP-1 RAs achieved a P-score of 0.89, SGLT2i a P-score of 0.57, and Placebo a P-score of 0.04 (**Figure 6**). Although the P-scores place GLP-1 RAs numerically ahead, the substantial overlap in confidence intervals of the individual treatment estimates and the indirect comparison HR means that the observed ranking represents a trend rather than definitive evidence of superiority (**Table 2**).

**Table 2:** Network meta-analysis and sensitivity analysis results for cardiovascular outcomes.

Outcome	Comparison	k (N)	NMA HR (95% CI) vs. Placebo	I²	Indirect Comparison SGLT2i vs GLP-1 RA HR (95% CI)	NMA p-value	Bucher p-value (sensitivity)	P-score (SGLT2i/GLP-1 RA)
MACE	SGLT2i vs Placebo	2 (8,509)	0.87 (0.77–0.97)	0%	1.05 (0.92–1.20)	0.44	0.48	0.57/0.89
	GLP-1 RA vs Placebo	5 (30,304)	0.83 (0.77–0.89)	0%				
HFH	SGLT2i vs Placebo	2 (8,509)	0.74 (0.62–0.90)	0%	0.83 (0.67–1.03)	0.09	0.08	0.94/0.50
	GLP-1 RA vs Placebo	4 (27,190)	0.90 (0.81–1.00)	0%				
MI	SGLT2i vs Placebo	1 (3,584)	0.78 (0.64–0.96)	—	1.05 (0.83–1.33)	0.68	0.70	0.58/0.87
	GLP-1 RA vs Placebo	3 (22,410)	0.74 (0.66–0.85)	0%				
CV Death	SGLT2i vs Placebo	2 (10,106)	0.98 (0.82–1.18)	0%	0.94 (0.76–1.16)	0.57	0.51	0.42/0.83
	GLP-1 RA vs Placebo	4 (25,593)	0.92 (0.83–1.02)	0%				

k, number of studies; N, total number of participants (active + placebo); NMA HR, hazard ratio from random-effects network meta-analysis; CI, confidence interval; I², heterogeneity; NMA p-value, two-sided p-value for the indirect comparison derived from the random-effects NMA model; P-score, frequentist ranking metric (1, best).

All indirect comparisons are derived from the star-shaped NMA. Bucher interaction p-values assess the original subgroup interaction between drug classes in a two-step meta-analysis. For MI and CV Death, the NMA estimates for SGLT2i are based on limited studies (1 and 2, respectively) and should be interpreted cautiously. The P-scores for CV Death (0.42 for SGLT2i, 0.83 for GLP-1 RA, 0.24 for Placebo) indicate a numerical ranking favouring GLP-1 RAs, but the wide confidence interval of the indirect comparison (0.76–1.16) and the limited SGLT2i evidence preclude any definitive conclusion of superiority.

This table summarizes the primary network meta-analysis (NMA) results together with sensitivity analyses. For each outcome, the table lists the number of studies (k) and total participants (N) contributing to each direct comparison versus Placebo, the pooled HR with 95% CI from the random-effects NMA, the I² statistic for heterogeneity, the indirect comparison HR (and 95% CI) between SGLT2i and GLP-1 RAs derived from the NMA, the Bucher interaction p-value (a subgroup interaction test comparing the two drug classes using original aggregate estimates, serving as a sensitivity analysis), and the P-scores for each class. P-scores are frequentist analogues of the surface under the cumulative ranking curve (SUCRA) and range from 0 to 1, where higher values indicate better efficacy in reducing events. For endpoints with only a single SGLT2i trial (MI), I² could not be calculated and ranking should be interpreted cautiously. For CV Death, the SGLT2i estimate is based on two trials (EMPACT-MI and DECLARE-TIMI 58), which remains limited. All indirect comparisons are exploratory as no head-to-head trials exist. HR, hazard ratio; CI, confidence interval; NMA, network meta-analysis; SGLT2i, sodium-glucose cotransporter-2 inhibitors; GLP-1 RA, glucagon-like peptide-1 receptor agonists.

#### 3.3.3 Sensitivity of the indirect comparison

The Bucher interaction test—comparing the two drug classes via subgroup analysis of the pooled HRs—yielded a non-significant p-value of 0.48, entirely consistent with the NMA indirect comparison. The original drug-class subgroup forest plots (**Supplementary Figure 10**) are now presented as a sensitivity analysis in the Supplementary Material.

Leave-one-out analysis for the NMA on MACE confirmed robustness: when any single study was removed, the pooled indirect comparison HRs varied between 0.83 and 0.85. A fixed-effect NMA model produced virtually identical results. Excluding all post-hoc subgroup-derived data (i.e., REWIND CVD subgroup) gave an HR of 0.855 (95% CI 0.803–0.911) in the combined analysis, closely matching the primary result.

### 3.4 Secondary outcome: heart failure hospitalization

#### 3.4.1 Combined class meta-analysis

The combined-class meta-analysis of the six studies reporting HFH data (2 SGLT2i, 4 GLP-1 RA) showed a significant overall reduction in heart failure hospitalisation (pooled HR 0.837, 95% CI 0.770–0.910; p < 0.001) (**Supplementary Figure 3**). Heterogeneity was again negligible (I² = 0.0%, 95% CI 0.0%–70.8%; τ² = 0.0000; Cochran’s Q p = 0.504). When separated by drug class, SGLT2i demonstrated a pooled HR of 0.76 (95% CI 0.65–0.90) and GLP-1 RAs a pooled HR of 0.91 (95% CI 0.83–0.99) (**Supplementary Figure 4**). The Bucher interaction test for the difference between these class-specific estimates gave a p-value of 0.084.

#### 3.4.2 Network meta-analysis

**Figure 7:**
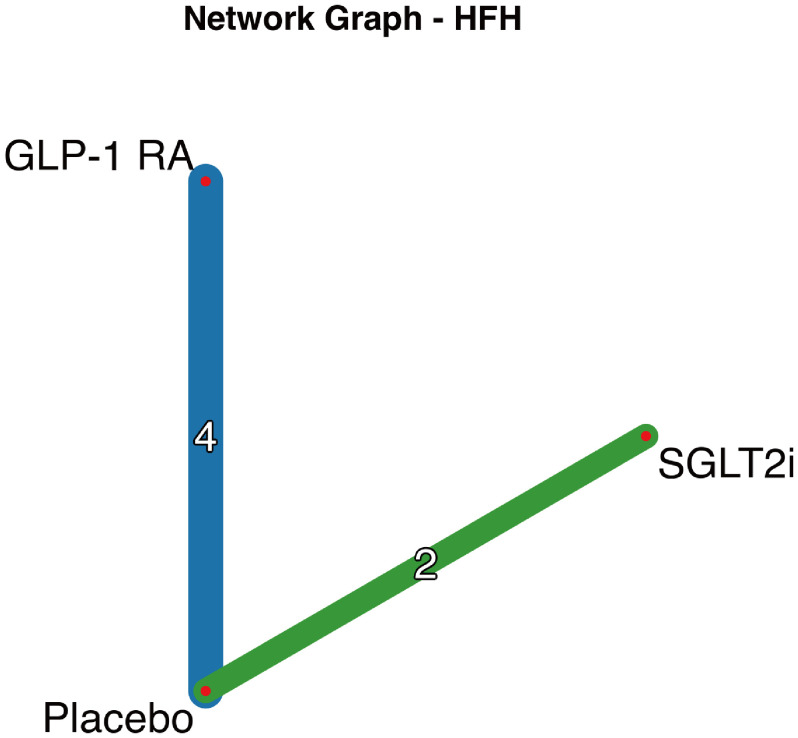
Network graph for heart failure hospitalization (HFH). Star-shaped evidence network for HFH (6 studies: 2 SGLT2i, 4 GLP-1 RA). Only active drug vs. Placebo comparisons are present.

**Figure 8:**
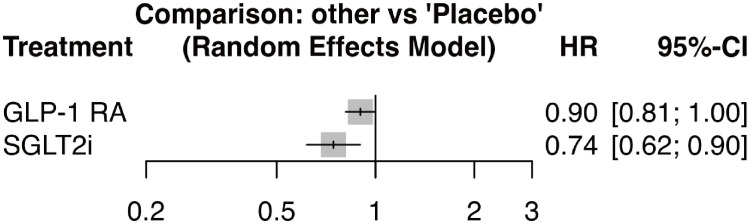
Network meta-analysis forest plot for heart failure hospitalization (HFH). Random-effects NMA estimates vs. Placebo. SGLT2i: HR 0.74 (95% CI 0.62–0.90); GLP-1 RA: HR 0.90 (95% CI 0.81–1.00). Heterogeneity: τ² = 0, I² = 0%.

The NMA for HFH comprised the same six studies and displayed a star-shaped network without closed loops (**Figure 7**). Under the random-effects NMA model, SGLT2i significantly reduced HFH compared to placebo (HR 0.74, 95% CI 0.62–0.90), whereas the reduction observed with GLP-1 RAs was smaller and its confidence interval approached the line of no effect (HR 0.90, 95% CI 0.81–1.00) (**Figure 8**). The indirect comparison between SGLT2i and GLP-1 RAs resulted in an HR of 0.83 (95% CI 0.67–1.03, p = 0.09).

**Figure 9:**
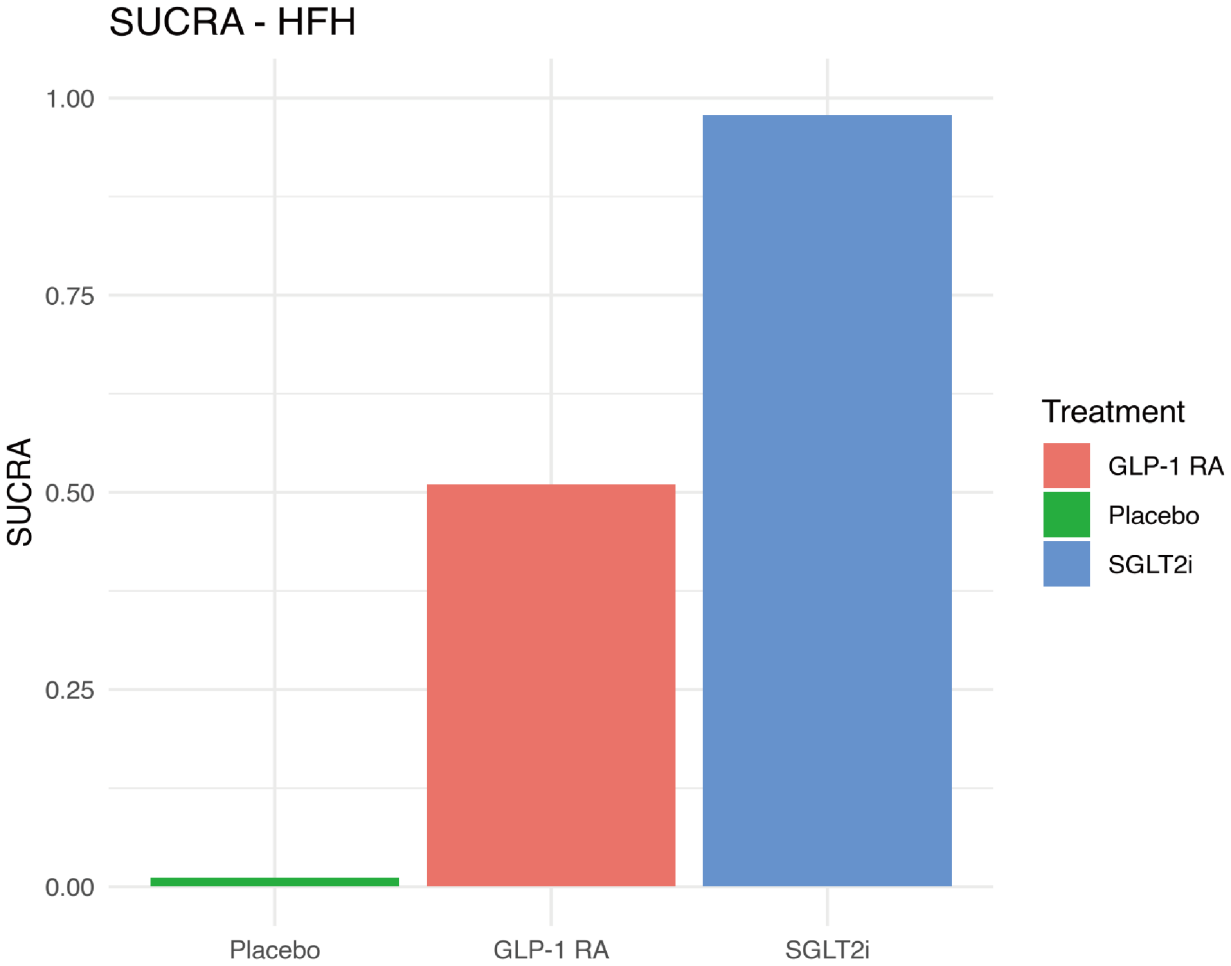
P-score ranking for heart failure hospitalization (HFH). Bar chart of P-scores. SGLT2i: 0.94; GLP-1 RAs: 0.50; Placebo: 0.06.

**Figure 10:**
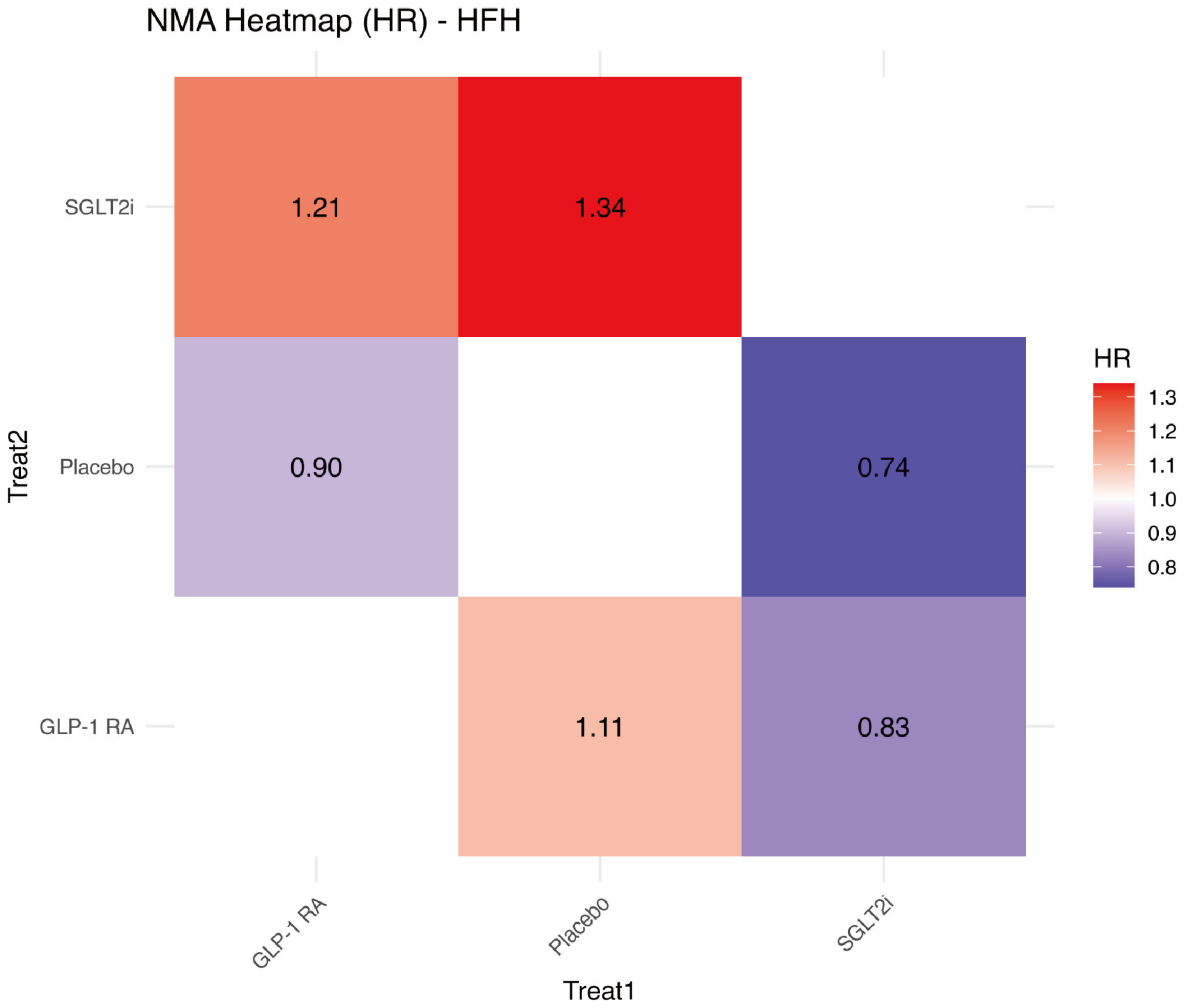
League table heatmap for heart failure hospitalization (HFH). Heatmap of pairwise NMA hazard ratios for HFH. Numerical HR values are overlaid on the coloured tiles.

The ranking analysis supported the visual impression from the forest plot: P-scores were 0.94 for SGLT2i, 0.50 for GLP-1 RAs, and 0.06 for Placebo (**Figure 9**). The league table heatmap (**Figure 10**) further illustrates the between-class difference, with a blue-shaded cell for the SGLT2i versus GLP-1 RA comparison indicating a favourable HR. The borderline interaction p-value from the Bucher test (p = 0.08) is consistent with this interpretation.

### 3.5 Secondary outcome: myocardial infarction

#### 3.5.1 Combined class meta-analysis

Four studies contributed data on recurrent myocardial infarction (1 SGLT2i, 3 GLP-1 RA). The combined-class random-effects meta-analysis showed a significant reduction in MI risk (pooled HR 0.755, 95% CI 0.681–0.838; p < 0.001) (**Supplementary Figure 5**). Heterogeneity was low (I² = 0.0%, 95% CI 0.0%–79.2%; τ² = 0.0000; Cochran’s Q p = 0.741). The Bucher interaction test comparing the two classes gave a p-value of 0.70, indicating no detectable difference. However, the ability to draw class-specific conclusions is severely limited by the fact that only one SGLT2i trial (DECLARE-TIMI 58) contributed to this outcome.

#### 3.5.2 Network meta-analysis

The NMA for MI is presented in **Supplementary Figures 6** (network), **S7** (forest), **S8** (P-scores), and **S9** (heatmap). The network geometry was star-shaped. In the NMA, GLP-1 RAs were associated with an HR of 0.74 (95% CI 0.66–0.85) versus placebo, and the single SGLT2i study produced an HR of 0.78 (95% CI 0.64–0.96). The indirect comparison between SGLT2i and GLP-1 RAs resulted in an HR of 1.05 (95% CI 0.83–1.33, p = 0.68), with the wide confidence interval encompassing both benefit and harm. The P-scores were 0.87 for GLP-1 RAs and 0.58 for SGLT2i, but given the sparse evidence for the latter, the ranking should be viewed as preliminary. While the point estimates suggest that both classes reduce MI risk, the indirect comparison does not support the superiority of one class over the other, and the data for SGLT2i are insufficient to make robust inferences.

### 3.6 Secondary outcome: cardiovascular death

Six studies contributed data on cardiovascular death: two SGLT2i trials (DECLARE-TIMI 58 and EMPACT-MI) and four GLP-1 RA trials (PIONEER 6, Harmony Outcomes, SOUL, and SUSTAIN-6). In the network meta-analysis (**Supplementary Figure 11**), GLP-1 RAs showed a non-significant trend toward reduced CV death compared with placebo (HR 0.92, 95% CI 0.83–1.02; p = 0.112), whereas SGLT2i demonstrated a neutral effect (HR 0.98, 95% CI 0.82–1.18; p = 0.860). The indirect comparison between SGLT2i and GLP-1 RAs yielded an HR of 0.94 (95% CI: 0.76–1.16, p = 0.57), with the confidence interval spanning both potential benefit and harm. The Bucher interaction test (**Supplementary Figure 13**) was consistent with the NMA, showing no statistically significant class difference (p = 0.51). Heterogeneity was minimal (I² = 0%, 95% CI: 0%–79.2%; τ² = 0).

Critically, the evidence for SGLT2i on CV death in this population is based on only two trials (DECLARE-TIMI 58 and EMPACT-MI), with DECLARE-TIMI 58 contributing a pre-specified MI subgroup analysis (HR 0.92, 95% CI 0.69–1.23). This limited evidence base precludes any firm conclusions regarding the comparative effect of SGLT2i and GLP-1 RAs on cardiovascular death. The wide confidence intervals and the neutral point estimate for SGLT2i underscore the need for larger, dedicated trials.

### 3.7 Subgroup analyses (original bucher interaction sensitivity analyses)

The pre-specified subgroup analyses—by timing of index MI and by GLP-1 RA administration route—were performed using the original Bucher interaction method and are retained as exploratory sensitivity analyses. These analyses were conducted exclusively on the MACE outcome using the same seven studies after exclusion of overlapping populations. The detailed results are presented in **Table 3**, and the corresponding forest plots are provided in **Supplementary Figures 14** and **S15**.

**Table 3:** Subgroup analyses for MACE.

Subgroup category	Subgroup	Studies (n)	Pooled HR (95% CI)	I² (%)	I² 95% CI
MI Timing	Chronic MI (>180 days)	2	0.81 (0.73–0.90)	0%	[0.0%; 73.2%]
	Recent MI (≤180 days)	1*	0.90 (0.76–1.06)	–	–
	Mixed/Unknown	4	0.86 (0.80–0.92)	0%	[0.0%; 73.2%]
GLP-1 RA Administration Route	Oral (PIONEER 6, SOUL)	2	0.85 (0.77–0.95)	0%	[0.0%; 84.2%]
	Subcutaneous (SUSTAIN-6, Harmony Outcomes)	2	0.74 (0.58–0.95)	0%	[0.0%; 84.2%]
	Other (REWIND CVD Subgroup)	1	0.87 (0.74–1.02)	–	–

The Recent MI category contained only EMPACT-MI; no pooled estimate was calculated.

Test for subgroup differences (MI timing): common effect p = 0.36, random effects p = 0.36.

Test for subgroup differences (GLP-1 RA route): p = 0.57.

MI timing subgroup analysis is based on the MACE outcome (7 studies). “Mixed/Unknown” refers to the REWIND CVD subgroup, for which individual patient-level time-since-MI data were not available. GLP-1 RA route of administration analysis is based on the five GLP-1 RA studies included in the MACE analysis (“Other” refers to the REWIND CVD subgroup, which used dulaglutide administered subcutaneously but with a different baseline population). All estimates are derived from random-effects models. Heterogeneity confidence intervals were calculated using the Q-profile method. MACE, major adverse cardiovascular events; MI, myocardial infarction; HR, hazard ratio; CI, confidence interval; I², measure of heterogeneity.

By timing of MI relative to randomization, studies were categorized into three groups: chronic MI (median time from MI to randomization >180 days), recent MI (≤180 days), and mixed/unknown (individual patient-level time data not available). For the chronic MI subgroup, which included two studies (Harmony Outcomes and DECLARE-TIMI 58), the pooled HR was 0.81 (95% CI 0.73–0.90) with no heterogeneity (I² = 0%). For recent MI, only one study (EMPACT-MI) was available, yielding an HR of 0.90 (95% CI 0.76–1.06). The confidence interval for this single-study estimate crossed the line of no effect, reflecting considerable statistical uncertainty. The mixed/unknown category, consisting of four studies (SUSTAIN-6, PIONEER 6, SOUL, and REWIND CVD Subgroup), gave a pooled HR of 0.86 (95% CI 0.80–0.92) with I² = 0%. The test for subgroup differences between the chronic and mixed/unknown subgroups was not significant (p = 0.36). Because only one study fell into the recent MI category, a formal overall interaction test across all three subgroups could not be meaningfully performed. The observed numerical point estimates—0.81, 0.90, and 0.86—suggest a consistent pattern of cardiovascular benefit regardless of the timing since MI, although the wider confidence interval in the recent MI subgroup underscores the lack of statistical power to detect effect modification. These findings should not be interpreted as evidence of diminished efficacy early after MI; rather, they reflect the sparse data available for the acute post-infarction period (**Supplementary Figure 14**).

By GLP-1 RA administration route, the five GLP-1 RA studies included in the MACE analysis were divided into three categories: oral semaglutide (PIONEER 6 and SOUL), subcutaneous GLP-1 RAs (SUSTAIN-6 and Harmony Outcomes), and the REWIND CVD Subgroup (dulaglutide, also administered subcutaneously but analyzed separately owing to its distinct trial population). The pooled HR for oral GLP-1 RAs was 0.85 (95% CI 0.77–0.95, I² = 0%), for subcutaneous GLP-1 RAs was 0.74 (95% CI 0.58–0.95, I² = 0%), and the single-estimate from the REWIND CVD Subgroup was 0.87 (95% CI 0.74–1.02). The test for subgroup differences yielded a p-value of 0.57, indicating no statistically significant variation among routes of administration. Although the point estimate for the subcutaneous group was numerically lower, the confidence intervals overlapped substantially, and the small number of studies in each subgroup prevents any definitive conclusion about differential efficacy by administration route. Overall, these findings suggest that the cardiovascular benefit of GLP-1 RAs in MACE reduction is robust and consistent regardless of whether the drug is given orally or subcutaneously (**Supplementary Figure 15**).

### 3.8 Sensitivity analyses

In addition to the NMA-based sensitivity analyses detailed in Sections 3.3–3.6, several pre-specified robustness checks were performed to evaluate the stability of the original meta-analytic approach.

First, a leave-one-out analysis for the combined-class MACE meta-analysis (seven studies) was conducted by iteratively removing each study and recalculating the pooled HR under a random-effects model. The resulting point estimates ranged from 0.830 (95% CI 0.778–0.886) when the REWIND CVD Subgroup was excluded, to 0.846 (95% CI 0.794–0.902) when DECLARE-TIMI 58 was excluded. In all iterations, heterogeneity remained at I² = 0%, and the 95% confidence interval consistently excluded the null value of 1.0. The full set of estimates is presented in **Table 4**. This analysis confirms that no single study exerted a disproportionate influence on the overall MACE result. Second, we evaluated the impact of post-hoc subgroup data by excluding all analyses that were derived from subgroups rather than from the primary trial populations. Specifically, when the REWIND CVD Subgroup was removed from the MACE meta-analysis, the pooled HR became 0.855 (95% CI 0.803–0.911), which is very close to the primary estimate of 0.838, supporting the robustness of our approach. Third, for all outcomes, we compared the primary random-effects models with fixed-effect models. Owing to the negligible between-study variance (τ² ≈ 0 across all analyses), the point estimates and confidence intervals were virtually identical under both models, confirming that the choice of meta-analytic model had no material impact on the conclusions. Fourth, the Bucher interaction tests (**Supplementary Figures 2**, **S4**) and the NMA indirect comparisons (**Figures 3**-**10**, **Supplementary Figures 6**-**S13**) provided completely concordant results: no statistically significant difference between SGLT2i and GLP-1 RAs was detected for any endpoint. This consistency across two different indirect comparison methodologies strengthens the credibility of the overall findings and supports the conclusion that the current evidence does not support the superiority of one drug class over the other for the outcomes examined.

**Table 4:** Sensitivity analysis for MACE (leave-one-out).

Excluded study	Pooled HR (95% CI)	I² (%)	Tau²
DECLARE-TIMI 58 (Dapagliflozin)	0.846 (0.794–0.902)	0%	0.0000
EMPACT-MI (Empagliflozin)	0.835 (0.784–0.889)	0%	0.0000
Harmony Outcomes (Albiglutide)	0.842 (0.790–0.899)	0%	0.0000
REWIND CVD Subgroup	0.830 (0.778–0.886)	0%	0.0000
SUSTAIN-6 (Subcutaneous Semaglutide)	0.842 (0.792–0.896)	0%	0.0000
PIONEER 6 (Oral Semaglutide)	0.841 (0.791–0.895)	0%	0.0000
SOUL (Oral Semaglutide)	0.833 (0.780–0.891)	0%	0.0000

The pooled HR from the main analysis (no study excluded) was 0.838 (95% CI: 0.789–0.890). Excluding any single study yielded pooled HRs ranging from 0.830 to 0.846, all with 95% confidence intervals excluding 1.0. Heterogeneity remained at I² = 0% in all iterations, indicating that no individual study disproportionately influenced the pooled estimate. HR, hazard ratio; CI, confidence interval; I², measure of heterogeneity.

The pooled HR (95% CI) from the main analysis (no study excluded) is 0.838 (0.789–0.890). Excluding any single study yielded pooled HRs ranging from 0.830 to 0.846, all with 95% confidence intervals excluding 1.0. Heterogeneity remained at I² = 0% in all iterations, indicating that no individual study disproportionately influenced the pooled estimate. HR, hazard ratio; CI, confidence interval; I², measure of heterogeneity.

### 3.9 Publication bias assessment

Publication bias was assessed separately for each of the four main outcomes (MACE, HFH, MI, and CV death). Because the number of studies contributing to each meta-analysis was fewer than ten, formal statistical testing using Egger’s regression test was not performed, as it would be underpowered and potentially misleading in this setting. Instead, we relied on visual inspection of funnel plots of the standard error of the log hazard ratio against the hazard ratio (**Supplementary Figure 16**). Across all four outcomes, the study-level estimates were distributed approximately symmetrically around the pooled effect size, without obvious gaps or pronounced skewness that would suggest suppression of small, non-significant studies. In particular, the funnel plot for MACE (**Supplementary Figure 16A**), which included the largest number of studies, showed no visual evidence of small-study effects. For HFH, MI, and CV death (**Supplementary Figures 16B–D**), the smaller number of studies limited the interpretability of the funnel plots, but no gross asymmetry was observed. Although these visual assessments cannot completely rule out publication bias or small-study effects, together with the comprehensive and systematic nature of our literature search, the consistency of effects across trials, and the low risk of bias in individual studies, publication bias is unlikely to have substantially influenced the overall results. This assessment was consistent with the GRADE evaluation, in which no downgrading for publication bias was applied.

## 4 Discussion

### 4.1 Principal findings and clinical implications

This indirect comparison meta-analysis, employing a formal network meta-analysis as the primary comparative method with Bucher interaction tests as sensitivity, provides evidence that both SGLT2 inhibitors and GLP-1 receptor agonists reduce the risk of MACE, HFH, and recurrent MI in patients with T2DM and a history of MI. The NMA confirmed the class-specific patterns suggested by the original pooled estimates: for MACE, GLP-1 RAs and SGLT2i showed comparable efficacy (indirect HR 1.05, 95% CI 0.92–1.20); for HFH, SGLT2i exhibited a numerically greater reduction (indirect HR 0.83, 95% CI 0.67–1.03), although the difference was not statistically significant. The observed effect sizes are clinically meaningful, especially given the high baseline cardiovascular risk in this population.

However, several fundamental limitations must be acknowledged upfront, as they govern the interpretation of all findings. First, no head-to-head randomized trials directly comparing SGLT2i and GLP-1 RAs exist. All comparisons between classes are indirect, derived from separate placebo-controlled trials using interaction tests on class-pooled estimates. Second, the majority of included studies enrolled patients with a median time since MI of several years (e.g., 5.4 years in DECLARE-TIMI 58), which does not align with the “recent MI” focus implied by the title. Our analysis therefore primarily applies to patients with a history of MI, with only limited data specifically addressing the acute post-infarction period (≤180 days). Third, the evidence base for cardiovascular death is extremely limited for SGLT2i (only two trials: EMPACT-MI and the pre-specified MI subgroup of DECLARE-TIMI 58), and for MI, only one SGLT2i trial contributed data. Fourth, the consistently observed I² = 0% point estimates are accompanied by wide 95% confidence intervals (e.g., 0%–70.8% for MACE), reflecting low statistical power to detect heterogeneity given the small number of studies; therefore, modest to substantial heterogeneity cannot be ruled out. These limitations are explicitly addressed throughout the discussion.

### 4.2 Interpretation of class-specific effects and comparison with existing literature

The formal network meta-analysis performed in this study provides the most statistically rigorous indirect comparison of SGLT2 inhibitors and GLP-1 receptor agonists in patients with type 2 diabetes and a history of myocardial infarction published to date. By synthesizing all available placebo-controlled evidence within a single model, the NMA yields more precise comparative estimates and permits ranking of treatments, while the Bucher sensitivity analyses confirm the robustness of the primary findings.

For the primary MACE endpoint, the indirect comparison produced an HR of 1.05 (95% CI 0.92–1.20), indicating that the two classes are statistically indistinguishable with respect to overall cardiovascular event reduction. The P-scores (0.89 for GLP-1 RAs vs. 0.57 for SGLT2i) hint at a numerically slightly greater MACE reduction with GLP-1 RAs, but the confidence interval of the indirect estimate comfortably includes 1.0, and the Bucher interaction test (p = 0.48) confirms the absence of a significant class difference. This finding is consistent with the original aggregate meta-analyses by Zelniker et al. and Kristensen et al., which reported broadly similar MACE effects for SGLT2i and GLP-1 RAs in broader populations of patients with type 2 diabetes and established cardiovascular disease or high cardiovascular risk (6, 7).

For heart failure hospitalization, the pattern was notably different. The NMA indirect comparison yielded an HR of 0.83 (95% CI 0.67–1.03), corresponding to a point estimate of a 17% relative risk reduction with SGLT2i over GLP-1 RAs. Although this did not reach conventional statistical significance (p ≈ 0.09 from the NMA, Bucher p = 0.084), the numerical advantage of SGLT2i is substantial and aligns with the well-recognized hemodynamic, natriuretic, and reverse-remodeling effects of this class (36–38). The P-score of 0.94 for SGLT2i versus 0.50 for GLP-1 RAs further underscores this signal. Our findings echo prior NMA-based comparisons conducted in broader populations, where SGLT2i also showed a larger HFH benefit, but our analysis uniquely confines this comparison to patients with prior MI—a group in whom heart failure risk is particularly elevated and in whom the absolute benefit of SGLT2i may therefore be greatest.

For myocardial infarction, both classes reduced the risk of recurrent MI substantially (GLP-1 RAs HR 0.74, SGLT2i HR 0.78 vs. placebo), and the indirect comparison (HR 1.05, 95% CI 0.83–1.33) again fails to detect a class difference. However, the interpretation is severely hampered by the fact that the SGLT2i MI estimate is driven entirely by a single trial (DECLARE-TIMI 58), a limitation shared by several previous meta-analyses (6). For cardiovascular death, the indirect comparison (HR 0.94, 95% CI 0.76–1.16) and the Bucher interaction test (p = 0.51) both fail to detect any class difference. The neutral effect of SGLT2i (pooled HR 0.98, 95% CI 0.82–1.18) is driven largely by the EMPACT-MI trial (HR 1.03, 95% CI 0.81–1.31) and the DECLARE-TIMI 58 MI subgroup (HR 0.92, 95% CI 0.69–1.23). Notably, the DECLARE-TIMI 58 data are derived from a pre-specified subgroup analysis, and the wide confidence intervals reflect considerable statistical uncertainty. The favourable but non-significant trend for GLP-1 RAs (HR 0.92, 95% CI 0.83–1.02) is consistent with prior meta-analyses (7). This trend may reflect their established effects on weight reduction, blood pressure lowering, lipid improvement, and direct anti-atherosclerotic properties. Given the sparsity of SGLT2i data, any comparative inference for this endpoint remains fragile and hypothesis-generating.

Our study adds distinct value to the existing literature in several respects. First, unlike prior comprehensive NMAs that included diverse populations across the cardiovascular risk spectrum (6, 7, 39), or focused on broader type 2 diabetes populations without restriction to prior MI (e.g., Palmer et al.’s study (40)), we deliberately restricted our analysis to patients with a history of MI. This narrow scope enhances clinical applicability for the post-MI population, in which residual risk is highest and in which cardiologists and endocrinologists most urgently seek comparative evidence. Second, we incorporated the latest trial evidence, notably the SOUL trial (oral semaglutide) and the full EMPACT-MI dataset, which had not been included in many earlier syntheses. Third, we carefully addressed the methodological challenge of overlapping populations—a pitfall that can lead to double-counting and inflated precision—by retaining only the REWIND CVD subgroup and the overall EMPACT-MI T2DM population, a refinement not uniformly applied in previous work. Fourth, the concordance between the NMA and the Bucher sensitivity analyses across all endpoints reinforces confidence in the robustness of our findings.

Nevertheless, our results must be placed in the context of large cardiovascular outcome trials in broader populations, such as EMPEROR-Reduced, DAPA-HF, and LEADER, which collectively demonstrate the robust benefits of both classes in reducing cardiovascular events. The present analysis should not be misconstrued as suggesting that one class is superior across all endpoints; rather, it suggests that for the specific post-MI population, clinicians may reasonably choose either class, while considering the numerically stronger HFH signal for SGLT2i and the potential for a more pronounced anti-atherosclerotic effect of GLP-1 RAs.

### 4.3 Subgroup analyses and heterogeneity

The subgroup analyses conducted as part of this review, although limited by the small number of available studies, offer clinically relevant insights and expose important evidence gaps. Subgroup analyses for MACE suggested consistent benefits across different timings of the index MI. In patients with chronic MI (>180 days from the event), the pooled HR was 0.81 (95% CI 0.73–0.90), derived from two large trials (Harmony Outcomes and DECLARE-TIMI 58). For recent MI (≤180 days), only the EMPACT-MI trial contributed data, yielding an HR of 0.90 (95% CI 0.76–1.06), which did not reach statistical significance and had a confidence interval that included 1.0. The mixed/unknown group, which comprised the remaining four studies, showed an intermediate benefit (HR 0.86, 95% CI 0.80–0.92). The overlap among these three point estimates (0.81, 0.90, and 0.86) and the non-significant test for subgroup differences (p = 0.36) suggest a generally consistent treatment effect, but the absence of a significant signal in the recent-MI subgroup should not be interpreted as evidence of reduced efficacy in the early post-infarction period. Rather, it reflects the very limited data: only one trial specifically enrolled patients in the acute phase after MI, and event rates in this single trial may have been insufficient to demonstrate a statistically robust effect. Future trials that explicitly target the immediate post-MI window and are adequately powered for subgroup interaction tests are needed to clarify whether the timing of drug initiation modifies the magnitude of benefit.

Regarding the GLP-1 RA administration route, we found no statistically significant difference between oral and subcutaneous formulations (p = 0.57). The point estimate for subcutaneous GLP-1 RAs (HR 0.74) was numerically lower than for oral semaglutide (HR 0.85), but this difference is based on only two studies per subgroup and could simply reflect the play of chance or minor differences in the baseline risk profiles of the trial populations. Moreover, the REWIND CVD Subgroup (dulaglutide, administered subcutaneously) exhibited an HR of 0.87, further tempering any simple inference about route-dependent efficacy. This finding aligns with the broader literature suggesting that the cardiovascular benefits of GLP-1 RAs are likely a class effect, mediated through metabolic improvements, weight loss, blood pressure reduction, and anti-inflammatory mechanisms, rather than being solely dependent on the route of administration.

Heterogeneity, as quantified by the I² statistic, was consistently 0% or very low across all primary NMA and pairwise meta-analyses, indicating that the included studies yielded remarkably concordant treatment effect estimates. Such uniformity strengthens confidence in the internal validity of the indirect comparisons and supports the assumption of transitivity that underlies NMA. However, the 95% confidence intervals for I² were wide (e.g., 0% to 70.8% for MACE, 0% to 79.2% for MI), reflecting the low statistical power to exclude moderate to substantial heterogeneity when the number of studies is small. As Higgins et al. have emphasized, I² estimates from small meta-analyses are imprecise, and a point estimate of 0% does not guarantee true homogeneity (24). Therefore, clinical heterogeneity—in terms of baseline patient characteristics, background medical therapy, duration of follow-up, and geographic setting—may still exist and should be considered when generalizing these findings to individual patients. Furthermore, the star-shaped network geometry of our NMA precluded formal testing of inconsistency between direct and indirect evidence, because there were no closed loops. This is an inherent limitation of indirect comparisons based solely on placebo-controlled trials. Despite these caveats, the low I², the consistency between the NMA and Bucher sensitivity analyses, and the robustness of the leave-one-out evaluations all suggest that the fundamental conclusions of this review are not driven by heterogeneity or inconsistency.

### 4.4 Safety outcomes

Safety outcomes (severe hypoglycemia, diabetic ketoacidosis, pancreatitis, serious adverse events) were pre-specified but could not be meta-analyzed quantitatively due to inconsistent definitions and reporting across trials. A narrative summary is provided in **Supplementary Table 2**. No major safety signals were identified in individual trials: severe hypoglycemia was not increased with either drug class; DKA was increased with dapagliflozin in DECLARE-TIMI 58 (HR 2.68, 95% CI: 1.30–5.50), consistent with the known class effect of SGLT2i; pancreatitis rates were not significantly elevated; and serious adverse event rates were comparable between active treatment and placebo. However, the absence of a quantitative synthesis limits the strength of safety conclusions, and rare adverse events may not be captured.

### 4.5 Strengths and limitations

This analysis has several notable strengths. First, it employed a comprehensive and systematic literature search spanning from database inception to February 2026, incorporating the most recent pivotal cardiovascular outcome trials (e.g., SOUL, EMPACT-MI). Second, and most importantly from a methodological standpoint, we supplemented the original Bucher interaction approach with a formal frequentist network meta-analysis (NMA). This NMA represents a substantial upgrade over simple subgroup interaction tests because it simultaneously synthesizes direct and indirect evidence within a unified model, yields more precise indirect effect estimates, and provides treatment rankings through P-scores. The inclusion of a star-shaped network, although precluding formal inconsistency assessment, allowed us to borrow strength across the entire body of evidence and to present a league table of all pairwise comparisons—an approach that aligns with current best practices for comparative effectiveness reviews. Third, rigorous steps were taken to avoid bias: duplicate independent screening and data extraction, use of the Cochrane RoB 2.0 tool, application of the GRADE framework, and a pre-registered PROSPERO protocol. Fourth, we carefully addressed the issue of overlapping populations by excluding the EMPACT-MI T2DM subgroup and the overall REWIND population while retaining the REWIND CVD subgroup, with sensitivity analyses confirming the robustness of this decision. Fifth, extensive sensitivity analyses—including leave-one-out evaluations, fixed-effect models, and the exclusion of post-hoc subgroups—consistently supported the stability of our main findings.

Nevertheless, several limitations must be carefully considered when interpreting the results. The most fundamental limitation is the absence of any head-to-head randomized trial directly comparing SGLT2 inhibitors and GLP-1 receptor agonists. All comparative statements between classes are therefore based on indirect evidence and should be regarded as hypothesis-generating rather than conclusive. The NMA, while more rigorous, cannot fully overcome the intrinsic biases of indirect comparisons, including differences in baseline populations, background therapies, and trial designs. For instance, the SGLT2i trials largely enrolled patients with a remote history of MI (median >5 years in DECLARE-TIMI 58), whereas the single dedicated post-MI SGLT2i trial (EMPACT-MI) had a shorter follow-up and was not specifically designed to compare drug classes. Second, the term “recent MI” in the title may be misleading because the evidence base overwhelmingly represents chronic, remote MI. Only EMPACT-MI and ELIXA included true acute coronary syndrome populations, and ELIXA did not contribute to the quantitative synthesis due to lack of extractable subgroup data. Thus, our conclusions primarily apply to patients with a history of MI rather than the immediate post-infarction period. Third, the evidence for CV death is limited, with only two SGLT2i trials contributing data (EMPACT-MI and the pre-specified MI subgroup of DECLARE-TIMI 58). Of these, DECLARE-TIMI 58 provided data from a subgroup analysis, which may introduce additional uncertainty. The wide confidence intervals for the indirect comparison (0.76–1.16) and the neutral point estimate for SGLT2i preclude any robust conclusion regarding the comparative effect on cardiovascular mortality. Similarly, the evidence for MI is also limited, with only one SGLT2i trial (DECLARE-TIMI 58) contributing to that endpoint. Fourth, although the point estimates of I² were 0% for most analyses, the 95% confidence intervals around I² were wide (e.g., 0%–70.8% for MACE), reflecting low statistical power to detect heterogeneity given the small number of studies. Therefore, moderate to substantial clinical or methodological heterogeneity cannot be excluded. Fifth, the star-shaped network geometry precluded a formal evaluation of inconsistency between direct and indirect evidence, which is an inherent limitation of all NMAs that rely solely on placebo-controlled trials. Sixth, safety outcomes could only be summarized narratively because of heterogeneous definitions and reporting across trials; thus, rare but serious adverse events may have been missed. Seventh, publication bias was assessed only by visual inspection of funnel plots, as the small number of studies per outcome rendered formal Egger’s tests underpowered. Finally, the lack of individual participant data prevented more granular subgroup analyses (e.g., by left ventricular ejection fraction, renal function, or exact time since the index MI).

Despite these limitations, the current analysis is, to our knowledge, the most methodologically stringent indirect comparison of SGLT2i and GLP-1 RAs specifically in patients with type 2 diabetes and a history of MI. The integration of NMA enhances the transparency and statistical efficiency of the comparative estimates and provides a clearer picture of the evidence gaps that future trials must address.

### 4.6 Implications for clinical practice and future research

The findings of this NMA carry direct implications for clinical decision-making. In the absence of head-to-head trials, both SGLT2 inhibitors and GLP-1 receptor agonists can be considered as part of secondary cardiovascular prevention in patients with T2DM and a history of MI. The lack of a statistically significant difference in MACE reduction (indirect HR 1.05, 95% CI 0.92–1.20) indicates that either class is a reasonable choice for overall cardiovascular protection. However, the numerically greater reduction in HFH with SGLT2i (indirect HR 0.83, 95% CI 0.67–1.03) suggests that these agents may be preferred in patients with a high baseline risk of heart failure or with established left ventricular dysfunction. Conversely, for individuals in whom atherosclerotic events (such as recurrent MI or stroke) are the predominant concern, GLP-1 RAs might be considered, given their slightly higher P-scores for both MACE and MI. Crucially, all these signals are derived from indirect comparisons and did not reach statistical significance at conventional thresholds; hence, clinical decisions should be primarily guided by individual patient characteristics, tolerability, cost, and local availability.

Current guidelines for acute coronary syndrome management emphasize the importance of initiating guideline-directed medical therapy, including glucose-lowering agents with proven cardiovascular benefit, prior to hospital discharge (8, 9). Our findings support the use of either SGLT2 inhibitors or GLP-1 receptor agonists in patients with T2DM and a history of MI. However, the evidence for the acute post-MI period (within 180 days) is scarce and largely limited to the EMPACT-MI trial. This underscores the need for future trials targeting the acute phase to provide definitive evidence that can directly inform discharge prescribing practices.

These results also highlight important evidence gaps that should shape future research agendas. First and foremost, dedicated head-to-head randomized controlled trials directly comparing an SGLT2 inhibitor with a GLP-1 receptor agonist in the post-MI population are urgently needed. Such trials should be adequately powered to detect differences not only in MACE but also in HFH, recurrent MI, and cardiovascular death. Second, future trials should systematically stratify patients by time since the index MI (e.g., acute vs. chronic) and by baseline heart failure status, to clarify whether the observed trends differ across clinical sub-phenotypes. Third, given the complementary mechanisms of action of these two drug classes, combination therapy (SGLT2i plus GLP-1 RA) merits dedicated investigation. If the benefits are additive or synergistic, combination therapy could become the new standard for secondary prevention in this exceptionally high-risk population. Fourth, better standardization of safety outcome definitions and reporting is required across CVOTs to enable robust quantitative synthesis of rare adverse events. Finally, real-world evidence studies and cost-effectiveness analyses will be essential to complement trial data and to inform clinical guidelines and health policy decisions regarding the optimal sequencing and accessibility of these therapies.

## 5 Conclusion

In patients with type 2 diabetes and a history of myocardial infarction, network meta-analysis of placebo-controlled trials suggests that both SGLT2 inhibitors and GLP-1 receptor agonists reduce MACE, HFH, and recurrent MI, with no statistically significant difference between classes in indirect comparisons. SGLT2i show a numerically greater HFH reduction, though this does not reach statistical significance. Evidence is drawn predominantly from remote MI populations and remains limited for CV death, where only two SGLT2i trials (EMPACT-MI and the DECLARE-TIMI 58 MI subgroup) contributed data. In the absence of head-to-head trials, all comparative findings are hypothesis-generating. Treatment selection should be individualized. Dedicated direct-comparison trials are urgently needed.

## Data Availability

The original contributions presented in the study are included in the article/**Supplementary Material**. Further inquiries can be directed to the corresponding author.

## Supplementary material

Supplementary Figure 1 Combined-class meta-analysis forest plot for MACE (sensitivity analysis). Random-effects meta-analysis of 7 studies comparing SGLT2i or GLP-1 RA vs. Placebo. Pooled HR: 0.84 (95% CI 0.79–0.89); I² = 0%.

Supplementary Figure 2 Subgroup forest plot for MACE by drug class (bucher interaction test). Class-specific pooled HRs: SGLT2i 0.88 (95% CI 0.79–0.98); GLP-1 RAs 0.84 (95% CI 0.79–0.89). Interaction p = 0.48.

Supplementary Figure 3 Combined-class meta-analysis forest plot for HFH (sensitivity analysis). Random-effects meta-analysis of 6 studies. Pooled HR: 0.87 (95% CI 0.80–0.94); I² = 0%.

Supplementary Figure 4 Subgroup forest plot for HFH by drug class (bucher interaction test). SGLT2i pooled HR: 0.76 (95% CI 0.65–0.90); GLP-1 RAs pooled HR: 0.91 (95% CI 0.83–0.99). Interaction p = 0.08.

Supplementary Figure 5 Combined-class meta-analysis forest plot for MI (sensitivity analysis). Random-effects meta-analysis of 4 studies. Pooled HR: 0.80 (95% CI 0.71–0.89); I² = 18.8%.

Supplementary Figure 6 Network graph for myocardial infarction (MI). Star-shaped network with 4 studies (1 SGLT2i, 3 GLP-1 RA).

Supplementary Figure 7 Network meta-analysis forest plot for myocardial infarction (MI). Random-effects NMA estimates vs. Placebo. GLP-1 RAs: HR 0.74 (95% CI 0.66–0.85); SGLT2i: HR 0.78 (95% CI 0.64–0.96). Only one SGLT2i trial contributed.

Supplementary Figure 8 P-score ranking for myocardial infarction (MI). P-scores: GLP-1 RAs 0.87; SGLT2i 0.58; Placebo 0.05.

Supplementary Figure 9 League table heatmap for myocardial infarction (MI).

Supplementary Figure 10 Network graph for cardiovascular death (CV death). Star-shaped network with 6 studies (2 SGLT2i, 4 GLP-1 RA).

Supplementary Figure 11 Network meta-analysis forest plot for cardiovascular death (CV death). Random-effects network meta-analysis estimates of each treatment compared with placebo for cardiovascular death. The analysis included 6 studies (2 SGLT2i trials: DECLARE-TIMI 58 and EMPACT-MI; 4 GLP-1 RA trials: PIONEER 6, Harmony Outcomes, SOUL, and SUSTAIN-6). Squares represent study-specific hazard ratios (HRs), with the size of each square proportional to the study’s weight in the random-effects meta-analysis. Horizontal lines indicate 95% confidence intervals (CIs). Diamonds represent the pooled NMA estimates. For SGLT2i versus placebo, the HR was 0.98 (95% CI: 0.82–1.18; p = 0.860); for GLP-1 RA versus placebo, the HR was 0.92 (95% CI: 0.83–1.02; p = 0.112). Heterogeneity was minimal (I² = 0%, 95% CI: 0%–79.2%; τ² = 0). The dashed vertical line at HR = 1 indicates the line of no effect. All comparisons are indirect as no head-to-head trials exist.

Supplementary Figure 12 P-score ranking for cardiovascular death (CV death). Bar chart of P-scores (frequentist analogues of the surface under the cumulative ranking curve, SUCRA) derived from the random-effects network meta-analysis. Higher P-scores indicate a greater probability of being the best treatment (i.e., most effective in reducing cardiovascular death). The P-scores were 0.83 for GLP-1 receptor agonists, 0.42 for SGLT2 inhibitors, and 0.24 for placebo. Although GLP-1 RAs ranked highest numerically, the wide confidence intervals of the indirect comparison (HR 0.94, 95% CI: 0.76–1.16; p = 0.57) and the limited SGLT2i evidence (only two trials) indicate that this ranking is exploratory and should not be interpreted as evidence of superiority.

Supplementary Figure 13 League table heatmap for cardiovascular death (CV death). Heatmap representation of the network meta-analysis league table displaying pairwise treatment comparisons as hazard ratios (HRs). The colour gradient indicates the direction and magnitude of effect: blue (HR < 1) favours the treatment in the row over the treatment in the column (protective effect); red (HR > 1) favours the comparator (harmful effect); yellow (HR = 1) indicates no difference. Numerical HR values are overlaid on the tiles. For the indirect comparison of SGLT2i versus GLP-1 RA, the HR was 0.94 (95% CI: 0.76–1.16), indicating no statistically significant difference between the two classes. Diagonal entries (treatment vs. itself) are left blank.

Supplementary Figure 14 Bucher interaction test for cardiovascular death (CV death) – sensitivity analysis. Forest plot of the Bucher interaction test comparing the two drug classes for cardiovascular death. The analysis was performed by first pooling SGLT2i trials (DECLARE-TIMI 58 and EMPACT-MI) and GLP-1 RA trials (PIONEER 6, Harmony Outcomes, SOUL, and SUSTAIN-6) separately using random-effects meta-analysis, followed by a subgroup interaction test to assess whether the treatment effect differed between classes. For SGLT2i versus placebo, the pooled HR was 0.97 (95% CI: 0.77–1.23); for GLP-1 RA versus placebo, the pooled HR was 0.92 (95% CI: 0.82–1.03). The test for subgroup differences yielded a p-value of 0.51, indicating no statistically significant interaction between drug classes. This sensitivity analysis is consistent with the primary network meta-analysis (indirect comparison HR 0.94, 95% CI: 0.76–1.16; p = 0.57). Squares represent study-specific HRs; diamonds indicate class-specific pooled estimates. Horizontal lines indicate 95% confidence intervals. The dashed vertical line at HR = 1 indicates the line of no effect.

Supplementary Figure 15 Subgroup forest plot for MACE (GLP-1 RAs) by administration route (bucher interaction). Oral: HR 0.85 (0.77–0.95); Subcutaneous: HR 0.74 (0.58–0.95). Interaction p = 0.57.

Supplementary Figure 16 Funnel plots for assessment of publication bias. Funnel plots for **(A)** MACE, **(B)** HFH, **(C)** MI, and **(D)** CV death. Standard error of the log hazard ratio is plotted against the hazard ratio. Visual inspection reveals no gross asymmetry, suggesting absence of substantial publication bias.

Supplementary File 1 PRISMA 2020 checklist.
