# Supplementary material for: Cardiovascular outcomes of SGLT2 inhibitors versus GLP-1 receptor agonists in patients with type 2 diabetes and a history of myocardial infarction: a systematic review and network meta-analysis of randomized controlled trials
Source: Front Endocrinol (Lausanne). 2026 Jul 29;17:1902802. doi: 10.3389/fendo.2026.1902802 (PMC13461399; doi:10.3389/fendo.2026.1902802)
Supplement: Supplementary file 1 [file SupplementaryFile1.docx]

| **Section and Topic** | **Item #** | **Checklist item** | **Location where item is reported** |
| --- | --- | --- | --- |
| **TITLE** | | |  |
| Title | 1 | Identify the report as a systematic review. | Page 1, Lines 1-4 |
| **ABSTRACT** | | |  |
| Abstract | 2 | See the PRISMA 2020 for Abstracts checklist. | Page 1-2, Lines 21-49 |
| **INTRODUCTION** | | |  |
| Rationale | 3 | Describe the rationale for the review in the context of existing knowledge. | Page 3-5, Lines 53-100 |
| Objectives | 4 | Provide an explicit statement of the objective(s) or question(s) the review addresses. | Page 5, Lines 92-99 |
| **METHODS** | | |  |
| Eligibility criteria | 5 | Specify the inclusion and exclusion criteria for the review and how studies were grouped for the syntheses. | Page 5-7, Lines 112-144 |
| Information sources | 6 | Specify all databases, registers, websites, organisations, reference lists and other sources searched or consulted to identify studies. Specify the date when each source was last searched or consulted. | Page 7, Lines 147-158 |
| Search strategy | 7 | Present the full search strategies for all databases, registers and websites, including any filters and limits used. | Page 7, Lines 147-158 |
| Selection process | 8 | Specify the methods used to decide whether a study met the inclusion criteria of the review, including how many reviewers screened each record and each report retrieved, whether they worked independently, and if applicable, details of automation tools used in the process. | Page 8, Lines 160-167 |
| Data collection process | 9 | Specify the methods used to collect data from reports, including how many reviewers collected data from each report, whether they worked independently, any processes for obtaining or confirming data from study investigators, and if applicable, details of automation tools used in the process. | Page 8, Lines 168-179 |
| Data items | 10a | List and define all outcomes for which data were sought. Specify whether all results that were compatible with each outcome domain in each study were sought (e.g. for all measures, time points, analyses), and if not, the methods used to decide which results to collect. | Page 6-7, Lines 129-138 |
|  | 10b | List and define all other variables for which data were sought (e.g. participant and intervention characteristics, funding sources). Describe any assumptions made about any missing or unclear information. | Page 8, Lines 168-179 |
| Study risk of bias assessment | 11 | Specify the methods used to assess risk of bias in the included studies, including details of the tool(s) used, how many reviewers assessed each study and whether they worked independently, and if applicable, details of automation tools used in the process. | Page 9, Lines 181-190 |
| Effect measures | 12 | Specify for each outcome the effect measure(s) (e.g. risk ratio, mean difference) used in the synthesis or presentation of results. | Page 9, Lines 197-198 |
| Synthesis methods | 13a | Describe the processes used to decide which studies were eligible for each synthesis (e.g. tabulating the study intervention characteristics and comparing against the planned groups for each synthesis (item #5)). | Page 10, Lines 200-201 & Page 11, Lines 225-237 |
|  | 13b | Describe any methods required to prepare the data for presentation or synthesis, such as handling of missing summary statistics, or data conversions. | Page 8, Lines 175-179 |
|  | 13c | Describe any methods used to tabulate or visually display results of individual studies and syntheses. | Page 9-11, Lines 197-206, 217-218 |
|  | 13d | Describe any methods used to synthesize results and provide a rationale for the choice(s). If meta-analysis was performed, describe the model(s), method(s) to identify the presence and extent of statistical heterogeneity, and software package(s) used. | Page 9-10, Lines 197-206 |
|  | 13e | Describe any methods used to explore possible causes of heterogeneity among study results (e.g. subgroup analysis, meta-regression). | Page 10, Lines 208-213 |
|  | 13f | Describe any sensitivity analyses conducted to assess robustness of the synthesized results. | Page 10, Lines 214-215 |
| Reporting bias assessment | 14 | Describe any methods used to assess risk of bias due to missing results in a synthesis (arising from reporting biases). | Page 10-11, Lines 220-224 |
| Certainty assessment | 15 | Describe any methods used to assess certainty (or confidence) in the body of evidence for an outcome. | Page 9, Lines 188-195 |
| **RESULTS** | | |  |
| Study selection | 16a | Describe the results of the search and selection process, from the number of records identified in the search to the number of studies included in the review, ideally using a flow diagram. | Page 11, Lines 280-297 |
|  | 16b | Cite studies that might appear to meet the inclusion criteria, but which were excluded, and explain why they were excluded. | Page 12, Lines 295-300 |
| Study characteristics | 17 | Cite each included study and present its characteristics. | Page 12-13, Lines 309-323 |
| Risk of bias in studies | 18 | Present assessments of risk of bias for each included study. | Page 13, Lines 325-337 & Figure 2 |
| Results of individual studies | 19 | For all outcomes, present, for each study: (a) summary statistics for each group (where appropriate) and (b) an effect estimate and its precision (e.g. confidence/credible interval), ideally using structured tables or plots. | Figures 3, 6, 8, 10 and Tables |
| Results of syntheses | 20a | For each synthesis, briefly summarise the characteristics and risk of bias among contributing studies. | Page 14, Lines 373-377; Page 15, Lines 405-407 |
|  | 20b | Present results of all statistical syntheses conducted. If meta-analysis was done, present for each the summary estimate and its precision (e.g. confidence/credible interval) and measures of statistical heterogeneity. If comparing groups, describe the direction of the effect. | Page 14-17, Lines 373-426, Table 2, Figures 3-11 |
|  | 20c | Present results of all investigations of possible causes of heterogeneity among study results. | Page 14, Lines 380-382; Page 15, Lines 411-412; Page 16, Lines 430-431; Page 17-18, Lines 447-464; Table 3; Figures 4,5,7,9,11-13 |
|  | 20d | Present results of all sensitivity analyses conducted to assess the robustness of the synthesized results. | Page 18, Lines 465-468 & Table 4 |
| Reporting biases | 21 | Present assessments of risk of bias due to missing results (arising from reporting biases) for each synthesis assessed. | Page 18-19, Lines 469-479 & Table 5 |
| Certainty of evidence | 22 | Present assessments of certainty (or confidence) in the body of evidence for each outcome assessed. | Page 13-14, Lines 326-374 & Table 1, Figure 2C |
| **DISCUSSION** | | |  |
| Discussion | 23a | Provide a general interpretation of the results in the context of other evidence. | Page 19-22, Lines 488-557 & Page 22-23, Lines 576-592 |
|  | 23b | Discuss any limitations of the evidence included in the review. | Page 23, Lines 593-599 |
|  | 23c | Discuss any limitations of the review processes used. | Page 23-24, Lines 600-611 |
|  | 23d | Discuss implications of the results for practice, policy, and future research. | Page 23, Lines 600-609 & Page 24-25, Lines 612-630 |
| **OTHER INFORMATION** | | |  |
| Registration and protocol | 24a | Provide registration information for the review, including register name and registration number, or state that the review was not registered. | Page 5-6, Lines 103-109 |
|  | 24b | Indicate where the review protocol can be accessed, or state that a protocol was not prepared. | Page 5-6, Lines 103-109 |
|  | 24c | Describe and explain any amendments to information provided at registration or in the protocol. | None |
| Support | 25 | Describe sources of financial or non-financial support for the review, and the role of the funders or sponsors in the review. | Page 27, Lines 874-877 |
| Competing interests | 26 | Declare any competing interests of review authors. | Page 27, Line 871 |
| Availability of data, code and other materials | 27 | Report which of the following are publicly available and where they can be found: template data collection forms; data extracted from included studies; data used for all analyses; analytic code; any other materials used in the review. | Page 26, Lines 865-869 |

*From:*  Page MJ, McKenzie JE, Bossuyt PM, Boutron I, Hoffmann TC, Mulrow CD, et al. The PRISMA 2020 statement: an updated guideline for reporting systematic reviews. BMJ 2021;372:n71. doi: 10.1136/bmj.n71

For more information, visit: <http://www.prisma-statement.org/>
