# Supplementary material for: Cardiovascular outcomes of SGLT2 inhibitors versus GLP-1 receptor agonists in patients with type 2 diabetes and a history of myocardial infarction: a systematic review and network meta-analysis of randomized controlled trials
Source: Front Endocrinol (Lausanne). 2026 Jul 29;17:1902802. doi: 10.3389/fendo.2026.1902802 (PMC13461399; doi:10.3389/fendo.2026.1902802)
Supplement: Supplementary file 2 [file Table1.docx]

| **Supplementary Table 1. Detailed Search Strategy for PubMed (conducted on February 1, 2026)** | |
| --- | --- |
| Step | Search Query |
| #1 | "Diabetes Mellitus, Type 2"[Mesh] OR "type 2 diabetes"[tiab] OR "T2DM"[tiab] OR "NIDDM"[tiab] OR "non-insulin-dependent diabetes"[tiab] |
| #2 | "Myocardial Infarction"[Mesh] OR "myocardial infarction"[tiab] OR "MI"[tiab] OR "heart attack"[tiab] OR "acute coronary syndrome"[tiab] OR "ACS"[tiab] OR "Percutaneous Coronary Intervention"[Mesh] OR "PCI"[tiab] OR "coronary artery disease"[tiab] |
| #3 | "Sodium-Glucose Transporter 2 Inhibitors"[Mesh] OR "SGLT2 inhibitor*"[tiab] OR "SGLT-2 inhibitor*"[tiab] OR "canagliflozin"[tiab] OR "dapagliflozin"[tiab] OR "empagliflozin"[tiab] OR "ertugliflozin"[tiab] OR "sotagliflozin"[tiab] |
| #4 | "Glucagon-Like Peptide-1 Receptor Agonists"[Mesh] OR "GLP-1 receptor agonist*"[tiab] OR "GLP-1 RA"[tiab] OR "liraglutide"[tiab] OR "semaglutide"[tiab] OR "dulaglutide"[tiab] OR "exenatide"[tiab] OR "lixisenatide"[tiab] OR "albiglutide"[tiab] |
| #5 | #3 OR #4 |
| #6 | "Randomized Controlled Trial"[Publication Type] OR "Controlled Clinical Trial"[Publication Type] OR "randomized"[tiab] OR "randomised"[tiab] OR "placebo"[tiab] OR "clinical trial"[tiab] |
| #7 | #1 AND #2 AND #5 AND #6 |
| #8 | #7 AND (2016/01/01:2026/02/01[pdat]) |
| **Final** | **#8, Limits: humans, English language** |
| **Notes**: This strategy was adapted for Embase (Emtree terms), Cochrane CENTRAL, and ClinicalTrials.gov. The search was performed from database inception to **February 1, 2026**. | |
