# Supplementary material for: Cardiovascular outcomes of SGLT2 inhibitors versus GLP-1 receptor agonists in patients with type 2 diabetes and a history of myocardial infarction: a systematic review and network meta-analysis of randomized controlled trials
Source: Front Endocrinol (Lausanne). 2026 Jul 29;17:1902802. doi: 10.3389/fendo.2026.1902802 (PMC13461399; doi:10.3389/fendo.2026.1902802)
Supplement: Supplementary file 3 [file Table2.docx]

| **Supplementary Table 2. Narrative Summary of Pre-specified Safety Outcomes** | | | | | |
| --- | --- | --- | --- | --- | --- |
| **Trial (Drug Class)** | **Severe Hypoglycemia** | **Diabetic Ketoacidosis (DKA)** | **Pancreatitis** | **Serious Adverse Events (SAEs)** | **Key Safety Finding** |
| **SGLT2 INHIBITORS** |  |  |  |  |  |
| DECLARE-TIMI 58 (Dapagliflozin) | No significant difference vs. placebo | **Higher with dapagliflozin** (HR 2.68, 95% CI 1.30-5.50) | No significant difference | Similar between groups (22.1% vs 22.6%) | DKA risk elevated but absolute rate low (~0.1%) |
| EMPACT-MI (Empagliflozin) | Not reported specifically for T2DM subgroup | Not reported separately | Not reported | Similar between groups (overall trial) | No new safety signals |
| **GLP-1 RECEPTOR AGONISTS** |  |  |  |  |  |
| ELIXA (Lixisenatide) | No significant difference | Not reported | No significant difference | Similar between groups | Well-tolerated |
| Harmony Outcomes (Albiglutide) | No significant difference | Not reported | No significant difference | Similar between groups | Well-tolerated |
| REWIND (Dulaglutide) | No significant difference | Not reported | No significant difference | Similar between groups | No increase in SAEs |
| SUSTAIN-6 (Semaglutide) | No significant difference | Not reported | No significant difference | Similar between groups | No increase in SAEs |
| PIONEER 6 (Oral Semaglutide) | No significant difference | Not reported | No significant difference | Similar between groups | No increase in SAEs |
| SOUL (Oral Semaglutide) | No significant difference | Not reported | No significant difference | Similar between groups | No increase in SAEs |
| **Overall Narrative Summary**:   - **Severe Hypoglycemia**: None of the included trials reported a statistically significant increase in severe hypoglycemia with either SGLT2i or GLP-1 RA compared to placebo. - **Diabetic Ketoacidosis (DKA)** : Only DECLARE-TIMI 58 provided detailed DKA data, confirming a known class effect of SGLT2 inhibitors (absolute rate increase ~0.06%). Other trials either did not report DKA systematically or had zero events. - **Pancreatitis**: No trial reported a significant increase in pancreatitis. - **Serious Adverse Events (SAEs)** : Overall SAE rates were comparable between active treatment and placebo groups across all trials.   **Limitations of this safety synthesis**: Safety outcome definitions and reporting methods varied considerably across trials. Quantitative meta-analysis was not feasible due to clinical and methodological heterogeneity. The absence of a reported difference does not confirm equivalence, particularly for rare events. | | | | | |
