# Supplementary material for: Cardiovascular outcomes of SGLT2 inhibitors versus GLP-1 receptor agonists in patients with type 2 diabetes and a history of myocardial infarction: a systematic review and network meta-analysis of randomized controlled trials
Source: Front Endocrinol (Lausanne). 2026 Jul 29;17:1902802. doi: 10.3389/fendo.2026.1902802 (PMC13461399; doi:10.3389/fendo.2026.1902802)
Supplement: Supplementary file 4 [file Table3.docx]

| **Supplementary Table 3. Baseline Characteristics of Included Randomized Controlled Trials** | | | | | | | | | | | |
| --- | --- | --- | --- | --- | --- | --- | --- | --- | --- | --- | --- |
| **Trial Name (Year)** | **ClinicalTrials.gov Identifier** | **Study Design** | **Intervention** | **Comparator** | **Relevant Population (T2DM + MI)** | **Sample Size (Relevant Cohort)** | **Mean Age (years)** | **Male (%)** | **Time Since Most Recent MI** | **Mean Follow-Up (years)** | **Outcomes Reported** |
| **SGLT2 Inhibitor Trials** |  |  |  |  |  |  |  |  |  |  |  |
| DECLARE-TIMI 58 (2019) [31] | NCT01730534 | Multicenter, double-blind, placebo-controlled RCT | Dapagliflozin 10 mg daily | Placebo | T2DM with established CVD including prior MI | 3,584 | 64 | 80% | Median 5.4 years (IQR 2.0-9.8) | 4.2 | MACE, MI, CV death^*^, HFH |
| EMPACT-MI (2024) [27, 29, 30, 34] | NCT04509674 | Multicenter, double-blind, placebo-controlled RCT | Empagliflozin 10 mg daily | Placebo | Patients with acute MI (within 14 days) with or without T2DM | 3,260 (T2DM subgroup) | 64 | 75% | Within 14 days | 1.8 | MACE, HFH, CV death |
| EMPACT-MI T2DM Subgroup (2025) [30] | NCT04509674 | Pre-specified subgroup analysis | Empagliflozin 10 mg daily | Placebo | T2DM patients with acute MI within 14 days | 1,200 (estimated) | 63 | 74% | Within 14 days | 1.8 | MACE, HFH, CV death |
| **GLP-1 Receptor Agonist Trials** |  |  |  |  |  |  |  |  |  |  |  |
| ELIXA (2023) [28] | NCT01147250 | Multicenter, double-blind, placebo-controlled RCT | Lixisenatide 10-20 μg daily | Placebo | T2DM with recent ACS (within 180 days) | 6,068 | 60 | 69% | Within 180 days (median 76 days) | 2.1 | MACE, CV death, HFH |
| Harmony Outcomes (2018) [25] | NCT02465515 | Multicenter, double-blind, placebo-controlled RCT | Albiglutide 30-50 mg weekly | Placebo | T2DM with CVD including prior MI | 9,463 | 64 | 69% | Not specified (stable CVD cohort) | 1.6 | MACE, MI, CV death, HFH |
| REWIND (2019) [26] | NCT01394952 | Multicenter, double-blind, placebo-controlled RCT | Dulaglutide 1.5 mg weekly | Placebo | T2DM with established CVD or CV risk factors | 9,901 | 66 | 53% | Not specified (prior MI in ~30%) | 5.4 | MACE, MI, CV death, HFH |
| REWIND CVD Subgroup (2019) [26] | NCT01394952 | Pre-specified subgroup analysis | Dulaglutide 1.5 mg weekly | Placebo | T2DM with established CVD including prior MI | 3,500 (estimated) | 67 | 70% | Not specified | 5.4 | MACE, MI, CV death, HFH |
| SUSTAIN-6 (2016) [32] | NCT01720446 | Multicenter, double-blind, placebo-controlled RCT | Subcutaneous Semaglutide 0.5-1.0 mg weekly | Placebo | T2DM with established CVD, CKD, or CV risk factors | 3,297 | 65 | 60% | Not specified (prior MI in ~60%) | 2.1 | MACE, MI, CV death |
| PIONEER 6 (2019) [33] | NCT02692716 | Multicenter, double-blind, placebo-controlled RCT | Oral Semaglutide 14 mg daily | Placebo | T2DM with established CVD or CKD | 3,183 | 66 | 68% | Not specified (prior MI in ~50%) | 1.5 | MACE, MI, CV death, HFH |
| SOUL (2025) [35] | NCT03914326 | Multicenter, double-blind, placebo-controlled RCT | Oral Semaglutide 14 mg daily | Placebo | T2DM with established CVD and/or CKD | 9,650 | 67 | 65% | Not specified (prior MI in ~55%) | 3.5 | MACE, MI, CV death |
| SELECT (2023) [3, 4] | NCT03574597 | Multicenter, double-blind, placebo-controlled RCT | Subcutaneous Semaglutide 2.4 mg weekly | Placebo | Overweight/obese patients with CVD but without diabetes | 17,604 | 62 | 72% | Not specified (prior MI in ~65%) | 3.3 | MACE (primary), MI, CV death |
| **Footnotes:**  **Abbreviations:** T2DM, type 2 diabetes mellitus; MI, myocardial infarction; CVD, cardiovascular disease; ACS, acute coronary syndrome; CKD, chronic kidney disease; RCT, randomized controlled trial; MACE, major adverse cardiovascular events (composite of cardiovascular death, non-fatal MI, or non-fatal stroke); CV death, cardiovascular death; HFH, heart failure hospitalization; IQR, interquartile range.  **Notes:**   1. Sample sizes for relevant cohorts represent the number of patients with T2DM and documented MI (recent or remote) included in the analyses for this meta-analysis. Exact numbers for some subgroups were estimated based on published percentages. 2. Time since most recent MI is reported as available from primary publications or supplementary appendices. "Not specified" indicates that the precise timing for the MI subgroup was not separately reported in the primary publication. 3. The SELECT trial enrolled patients without diabetes but with established CVD including prior MI; it was included for its MI subgroup data relevant to GLP-1 RA cardiovascular effects in post-MI populations. 4. Multiple publications from the same trial (e.g., EMPACT-MI) are cited collectively under the primary trial name for simplicity. 5. * The CV death data for DECLARE‑TIMI 58 were derived from the pre‑specified MI subgroup analysis, as reported by Furtado et al. (Circulation 2019; 139:2516–2527). This subgroup analysis provided the HR of 0.92 (95% CI 0.69–1.23) for cardiovascular death in patients with prior MI, which was included in the CV death NMA for SGLT2i. | | | | | | | | | | | |
